# Infectious keratoconjunctivitis in semi-domesticated reindeer (*Rangifer tarandus tarandus*): a questionnaire-based study among reindeer herders in Norway and Sweden

**DOI:** 10.1186/s13028-023-00694-x

**Published:** 2023-07-12

**Authors:** Karin Wallin Philippot, Jerome Baron, Javier Sánchez Romano, Heidi Rautiainen, Jenny Frössling, Ingebjørg Helena Nymo, Ylva Persson, Anna Omazic, Morten Tryland

**Affiliations:** 1 Department of Animal Health and Antimicrobial Strategies, National Veterinary Institute, 751 89 Uppsala, Sweden; 2 Department of Clinical Sciences, Faculty of Veterinary Medicine and Animal Science, Swedish University of Agricultural Sciences, 750 07 Uppsala, Sweden; 3 Department of Disease Control and Epidemiology, National Veterinary Institute, 751 89 Uppsala, Sweden; 4 Present Address: Heidelberg Institute of Global Health, Faculty of Medicine at Heidelberg University, 69120 Heidelberg, Germany; 5 Vascular Biology Research Group, Department of Medical Biology, Faculty of Health Sciences, UiT - The Arctic University of Norway, 9037 Tromsø, Norway; 6 Department of Animal Nutrition and Management, Faculty of Veterinary Medicine and Animal Science, Swedish University of Agricultural Sciences, 750 07 Uppsala, Sweden; 7 Department of Animal Environment and Health, Swedish University of Agricultural Sciences, 532 23 Skara, Sweden; 8 Research Group Food Safety and Animal Health, The Norwegian Veterinary Institute, 9016 Tromsø, Norway; 9 Department of Arctic and Marine Biology, Faculty of Biosciences, Fisheries and Economics, UiT - The Arctic University of Norway, 9037 Tromsø, Norway; 10 Department of Chemistry, Environment and Feed Hygiene, National Veterinary Institute, 751 89 Uppsala, Sweden; 11 Department of Forestry and Wildlife Management, Inland Norway University of Applied Sciences, 2480 Koppang, Norway

**Keywords:** Climate change, Eye disease, Free-range, Infectious disease, IKC, Natural pastures, Reindeer husbandry, Supplementary feeding

## Abstract

**Background:**

The effects of climate change, loss of pastureland to other land usage and presence of large carnivores are the main reasons for the increase in supplementary feeding of semi-domesticated reindeer (*Rangifer tarandus tarandus*) in Fennoscandia over the last decades. Feeding might expose reindeer to stress and increased animal-to-animal contact, leading to an increased risk of infectious disease transmission, such as infectious keratoconjunctivitis (IKC). As it can develop rapidly and be very painful, IKC is described as an important animal welfare concern and a potential source of economic loss. The aim of this study was to investigate the current presence of IKC and potential associations between IKC and supplementary feeding through an online questionnaire survey, distributed among reindeer herders in Norway and Sweden in 2021.

**Results:**

Seventy-six reindeer herders (33 from Norway and 43 from Sweden) responded to the questionnaire, representing 6% and 4% of the registered reindeer herding groups in Norway and Sweden, respectively. Infectious keratoconjunctivitis was common, with 54 (71%) of the 76 herders that responded having observed clinical signs during the past 10 years. These signs were mainly observed as increased lacrimation, causing “wet cheeks”, but also as keratitis and conjunctivitis. Autumn and winter were the seasons in which IKC was observed most. The herders reported several measures, such as slaughter and isolation of affected reindeer, to counteract the spread of disease. The herding year 2019/2020 was associated with reports of outbreaks of IKC in herds as well as being the herding year where most herders (80%) had performed supplementary feeding. A significant association was found between IKC and feeding performed in an enclosure (odds ratio = 15.20), while feeding on free-range areas had a non-significant, negative, relationship with the appearance of IKC outbreaks (odds ratio = 0.29). Finally, there was a trend in the data suggesting that IKC affected calves especially.

**Conclusions:**

Infectious keratoconjunctivitis is a common disease, mainly observed in winter and autumn. It usually has mild to moderately severe clinical signs. Our results imply that IKC is associated with stress and feeding situations and that calves might be more susceptible than adults, however, this needs to be confirmed with further studies, preferably at an individual animal level.

**Supplementary Information:**

The online version contains supplementary material available at 10.1186/s13028-023-00694-x.

## Background

Semi-domesticated Eurasian tundra reindeer (*Rangifer tarandus tarandus*) in Norway and Sweden are mostly free-ranging and dependent on access to natural pastures. Ongoing climate change is causing a warmer climate with an increase in precipitation and fast changes in snow depth. This has resulted in winters with more frequent rain-on-snow and freeze–thaw events, causing ice-locked, inaccessible pastures, challenging the traditional methods used in reindeer husbandry [1–5]. The effects of climate change in combination with the loss of pastureland due to other land uses (forestry, infrastructure, mining, wind power parks, and tourism) and the presence of large carnivores (e.g., wolves, bears, lynxes, wolverines and eagles) are the main reasons for the increased need for supplementary feeding, in natural pastures or full feed rations in enclosures, given over several weeks or months in the wintertime seen in Fennoscandia over recent decades [6, 7].

In Norway and Sweden there are different types of reindeer herding districts, based on migration patterns and geography. In Sweden, there are three types of districts; mountain, forest, and concession herding districts. Mountain herding districts are the dominant form in both countries, whereas forest herding districts only exist in Sweden. The Swedish Sámi concession herding district resembles the forest district type with limited seasonal migration restricted to the boreal forest year around. The right to reindeer husbandry is an exclusive right to the Sámi people in both countries. However, special legislation allows non-Sámi ownership in the concession areas [8].

The present use of supplementary feeding and general feeding practices differs between countries and regions in Fennoscandia, and it varies considerably between years. Emergency feeding, to prevent starvation, is practiced in all countries, while regular feeding for several months is mostly practiced in the southern parts of the reindeer herding districts in Finland; it occurs only sporadically in the northern parts of reindeer herding districts in Finland and in Norway and Sweden. In addition, temporary feeding during gathering and migration periods has been commonly practiced for decades in areas in Sweden but does not seem as common in Norway. Grass silage, hay, and grain-based pellets are commonly used [6, 9–12]. The winter season during the herding year 2019/2020 was reported by many herders in Fennoscandia as being very challenging with ice crust formation in the snow, ice layers on the ground, and deep snow leading to unavailable winter pastures. Because of this, many herders had to implement supplementary feeding [6].

Supplementary feeding is conducted either by bringing the feed to the reindeer pastures or by feeding the animals in enclosures [6]. Gathering and feeding might expose the animals to stress, which could negatively affect the immunological status of the animals and increase their susceptibility to infections. In addition, intensified herding associated with supplementary feeding will often increase animal-to-animal contact and may contribute to unfavourable hygienic conditions on feeding spots, with an increased risk of infectious disease transmission [2, 13–16]. It is also known that supplementary feeding is associated with increased risk of feeding related disorders, changes in reindeer behaviour, as well as increased financial burden and workload for the reindeer herder.

A disease with a major impact on animal welfare in reindeer husbandry is infectious keratoconjunctivitis (IKC) (“Čalbmevikke” in Sámi), which is a multifactorial disease that has been known for more than a century [17, 18]. Regional and seasonal differences in management have been described as contributing factors in previous studies [19, 20]. Stress associated with herding, gathering, transport, and translocation to new environments, as well as environmental conditions and management factors, has been associated with large outbreaks of IKC, primarily affecting calves and yearlings. Recently, cervid herpesvirus 2 (CvHV2) was established to be a primary pathogen for IKC [21–24]. The virus remains latent in the animal after infection and is enzootic in most reindeer populations in Fennoscandia, with a typically higher prevalence of antibodies in adults than in calves [25–30]. Other microbiological agents, like *Chlamydia* spp*.*, *Moraxella bovoculi, Listeria monocytogenes*, and Pestivirus, have been identified as possible causes or co-infections contributing to IKC [14, 21, 22, 31]. Due to CvHV2’s ability to cause mucosal lesions in the eye and mouth, CvHV2 can facilitate secondary infections caused by other pathogens, as well as co-infections [32]. An additional pathogen, enzootic in Norway and Finland, that could have a pathological impact and may potentially present ocular signs in reindeer is gammaherpesvirus (malignant catarrhal fever virus group; MCFV) [25, 33], but the clinical impact of this virus in reindeer needs to be investigated further. Infectious keratoconjunctivitis severely affects animal welfare as the condition is very painful and follows a potentially rapid course which could lead to blindness. Furthermore, this causes difficulties for the reindeer when following the herd and finding feed, and, consequently, can lead to stress, potential starvation, and an increased risk of injury or death by predators. Non-infectious causes, such as insect bites and trauma, can give rise to clinical signs similar to IKC (i.e., keratitis, conjunctivitis, increased lacrimation) but are unlikely to cause outbreaks of IKC.

Tryland et al. [34] conducted a questionnaire survey around 10 years ago. The questionnaire was distributed among reindeer herders in Norway and Sweden and revealed that IKC was common in reindeer. Since then, climate change and the restricted availability of reindeer pastures, combined with economic support for feeding as compensation, has led to an additional increase in supplementary feeding in Norway and Sweden [6, 7, 14]. This may have potential effects on health and the transmission of infectious diseases, such as IKC, and an updated and extended investigation of the herders’ perspectives, traditions, and experiences over the recent decade was warranted. Hence, the objective of this study was to obtain an overview of the current presence of IKC in semi-domesticated reindeer in Norway and Sweden, and the potential association between IKC and supplementary feeding. Our hypothesis was that occurrence of IKC is associated with supplementary feeding practices.

## Methods

### Questionnaire survey

The study was conducted as a questionnaire survey. A link to a digital questionnaire together, with information about the study, was distributed by e-mail to a contact person for each herding district (siida/sijte) in Norway (n = 82) and to the chairperson of each herding district (Sameby) in Sweden (n = 51). The contact person and chairperson for each district was further urged to distribute and invite all their members to the questionnaire. In 2020, 535 and 1048 reindeer herding groups were registered in Norway (siida-shares) and Sweden (usually representing a family), respectively, meaning a total of 1583 active groups of reindeer herders could be reached. In addition, the questionnaire was accessible through the Sámiid Riikkasearvi’s (Sámi Reindeer Association, SSR) webpage to the members of 44 herding districts and 17 Sámi associations, and members of one private Facebook group (n = 87) engaged in reindeer health. The survey was accessible from the 16th of April 2021 in Norway, and from the 26th of April 2021 in Sweden, until the 6th of September 2021 for both countries. Monthly reminders were sent out by e-mail. In Sweden, the chairperson of each herding district was also reminded by phone. All the respondents in this study are hereafter referred to as herders. Information regarding the handling and storage of data and written informed consent was provided to all respondents at the start of the questionnaire. In addition, a permit from the Norwegian Social Science Data Services was obtained for the collection and handling of personal data (Approval No. 457339). Similar declarations were not required in Sweden, based on instructions from the Swedish Ethical Review Authority.

### Development of the questionnaire

The questionnaire was available in Swedish, Norwegian and Northern Sami (Additional files 1, 2, 3). It was generated in the web-based service Questback Essentials (Questback Sweden Ltd., Stockholm, Sweden, version number 38, 2021). The questionnaire contained 139 questions of various types, including simple yes/no and multiple-choice questions, and some supplemented with images. A few mandatory questions, mainly at the start of each section, were followed by questions based on the answer to the previous question (conditional branching). The respondents were able to add comments continuously. The questions referred to observations made over the past five or 10 years, or during the herding year 2019/2020. The questionnaire was divided into six sections: demographics, IKC, contagious ecthyma, oral necrobacillosis, other diseases, and feeding. This study has focused on the responses to questions from three parts, e.g., demographics, IKC, and parts of the feeding section.

### Demographics and herd data

The first part of the questionnaire concerned herd data, including country, region, and herd size (divided into eight intervals from < 50 to > 3000, Additional files 1, 2, 3). For Sweden, the type of reindeer herding district, i.e., mountain, forest, and concession herding district, was also included. Demographic data included gender and age.

### Presence and clinical signs of infectious keratoconjunctivitis

**Fig. 1:**
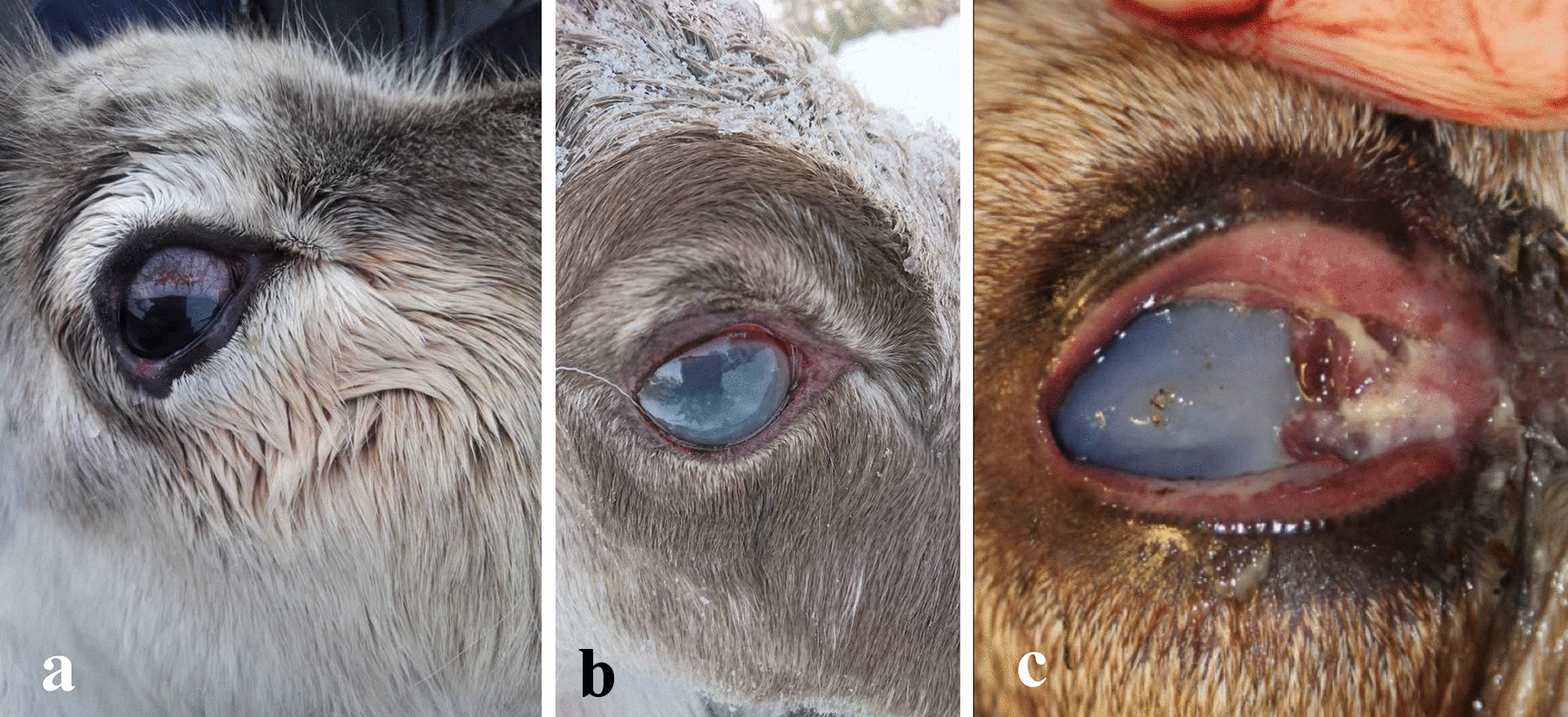
Images showing the clinical signs of infectious keratoconjunctivitis (IKC) in reindeer used in the questionnaire distributed to Norwegian and Swedish reindeer herders. **a** Increased lacrimation (epiphora) causing “wet cheeks”, which are a sign of the early and mild stage of IKC. **b** Conjunctivitis and corneal oedema, indicated as bluish/whitish discoloration of the cornea, which are both typical clinical signs of IKC at a moderately severe stage. **c** A very severe and often irreversible stage of IKC, including clinical signs like peri-orbital oedema and swelling (chemosis) and shedding of pus. Images from: Sámiid Riikkasearvi (Sámi Reindeer Association) and Farm and Animal Health’s image archive

In the second part of the questionnaire, three images of different clinical stages of IKC were presented (Fig. 1). The questions addressed whether similar clinical signs had been noticed over the last 10 years, and the respondent’s level of certainty related to such observations. Further questions on this topic were: when IKC was last observed; the most common clinical signs observed; the season of the year when clinical signs were observed; and the age category, calf (< 1 year old), young (1–3 years old), and adult (> 3 years old), and number of reindeer with IKC. We also asked if the respondents had experienced an outbreak of IKC. In the questionnaire, an outbreak was defined as an evident increase in the number of clinical cases observed over a period of time, it could be over longer (season) or shorter (few weeks) periods of time. Furthermore, questions about herding year and herding conditions, e.g., supplementary feeding or not, and if so, whether this was in an enclosure or free-range, were asked. This was followed by questions about any observed changes in the presence of the disease over the past 5 years, and for what reasons, followed by questions regarding what management interventions were used when IKC was last observed. We also asked if the respondents had experienced economic consequences in relation to IKC (e.g., loss of reindeer, increased workload or veterinary expenses), and if they had any traditional name or traditional knowledge associated with the disease.

### Supplementary feeding

In the third part of the questionnaire, the use of supplementary feeding was addressed. Feeding was defined as giving supplementary feed to a winter herd for more than 2 weeks. The feeding section of the questionnaire was divided into the following four sections: (1) A general part, involving when and where (enclosure and/or free-range) feeding took place over the past five herding years; (2) The reason why feeding was conducted; (3) The effects of feeding; and (4) The detailed feeding routines for the herding year 2019/2020 and general feeding routines over the past 5 years. From this part of the questionnaire, only certain data from the general questions (section 1) are presented in this study.

### Data management and statistical analysis

Initial exploratory data analysis was performed using Questback Essentials (Questback Sweden Ltd., Stockholm, Sweden, version number 38, 2021). Further data management, maps and graphs were produced and analysed in R, version 4.1.0 (R Core Team, 2021), and demographic data was calculated as percentages and presented in tables. The quotations presented in the results are based on written comments from the respondents and are thus not verbatim, but the meaning and essence of the respondents’ words have not been altered.

To evaluate the potential association between supplementary feeding and the appearance of IKC, mixed effect logistic regression, using the “lme4” package was done on survey questions that covered multiple herding years, from 2015–2016 to 2020–2021. In each regression model, the outcome variable was the observed presence of an IKC outbreak in the herd for a given herding year (yes or no; 1/0), with each observation under analysis being a herding year per herd. The exposure variables of interest were three binary dummy variables related to feeding. The first two dummy variables were feeding in an enclosure and free-range feeding. The third dummy variable, feeding overall, is a variable created based on the two prior ones (feeding in an enclosure and free-range feeding). These were investigated in separate univariable models. Herd ID was used as a random effect to account for the repeated nature of this data over time, and thus the lack of independence between observations from the same herd at different points in time. A simple multivariable model selection was conducted considering these main exposures as well as some spatiotemporal and demographic covariates (herd size, region, country, and herding year). Since several Norwegian counties had very few herds surveyed, these were aggregated for modelling, with Nord-Trøndelag, Sør-Trøndelag, and Hedmark forming one region, and Nordland and Troms forming another. Model selection was done in a two-way approach, starting from an empty model and using the Akaike Information Criterion (AIC) with a likelihood ratio test (LRT). Given the small sample size, a *P*-value of 0.1 was used on the LRT to include a variable in the final model. Similarly, *P* = 0.1 was used to identify a significant association, with *P* ≤ 0.05 being considered “significant” and *P* > 0.05 and < 0.1 being considered “borderline significant”.

## Results

### Demographics, herding district, and herd data

**Fig. 2:**
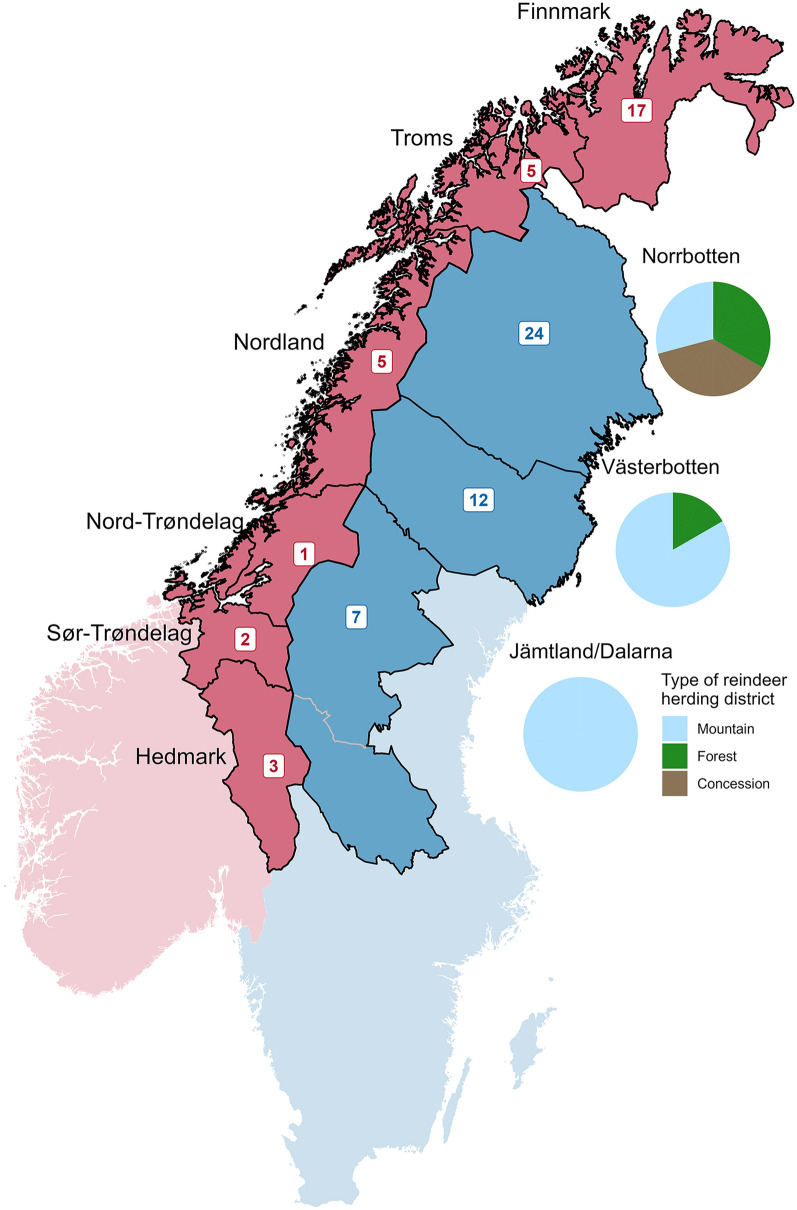
Number of respondents to the questionnaire survey regarding the health and supplementary feeding of semi-domesticated reindeer, divided per county, Norway (in red; designated as before 2020 when several counties were merged) and Sweden (in blue; where Jämtland and Dalarna are merged). The pie charts show the distribution of type of reindeer herding district (mountain, forest, and concession districts) in the four Swedish counties

In total, 134 herding district representatives in Norway and Sweden received the link to the questionnaire, and 76 reindeer herders responded to the questionnaire, 33 from Norway and 43 from Sweden (Table 1), representing 6% and 4% of the registered reindeer herding groups in Norway and Sweden, respectively. Responses were received from all reindeer herding regions, except for the county of Møre og Romsdal in Norway, which is hosting only a restricted number of semi-domesticated reindeer. In Sweden, nine respondents were from concession districts (21%), 10 from forest districts (23%), and 24 from mountain districts (56%, Fig. 2). The distribution of gender, age and herd size are presented in Table 1. The herd size distribution varied both between, and within the countries (Table 1, Additional file 4).

**Table 1:** General demographic data for the 76 herders that responded to the questionnaire survey distributed in Norway and Sweden in 2021 regarding the health and supplementary feeding of semi-domesticated reindeer

Parameter	Category	Norway n (%^a^)	Sweden n (%^a^)	In total n (%^a^)
Respondents	In total	33 (43)	43 (57)	76 (100)
Gender	Female	6 (18)	21 (49)	27 (36)
	Male	27 (82)	19 (44)	46 (61)
	Unspecified	0 (0)	3 (7)	3 (4)
Age (years)	< 20	0 (0)	0 (0)	0 (0)
	20–39	9 (27)	13 (30)	22 (29)
	40–59	19 (58)	24 (56)	43 (57)
	> 60	5 (15)	6 (14)	11 (14)
Herd size	1–499	13 (39)	10 (23)	23 (30)
	500–999	6 (18)	11 (26)	17 (22)
	1000–1999	4 (12)	12 (28)	16 (21)
	> 2000	10 (30)	10 (23)	20 (26)

^a^Since decimals were omitted, the sum is not necessarily 100 (%) for each parameter

### Presence and clinical signs of infectious keratoconjunctivitis

In total, 51 of the 76 respondents (67%) had observed clinical signs of IKC over the past 10 years, whereas three (4%) answered that they were unsure or had seen other clinical signs than presented in Fig. 1. Results per country show that 20 (61%) of the 33 respondents in Norway and 34 (79%) of the 43 respondents in Sweden had observed IKC over the past 10 years. Most of the respondents had observed clinical signs of IKC in the last year (Table 2).

**Table 2:** General distribution of answers related to when infectious keratoconjunctivitis was last observed over the past 10 years, from 54 herders surveyed in the questionnaire regarding health and supplementary feeding of semi-domesticated reindeer in 2021, with 20 and 34 respondents from Norway and Sweden, respectively

Question	Category	Number and frequency^a^ of answers		
		Norway	Sweden	Total
When did you last observe eye disease?	Last year	11 (55)	23 (68)	34 (63)
	Not last year, but in the last 5 years	5 (25)	6 (18)	11 (20)
	> 5 years ago	3 (15)	2 (6)	5 (9)
	Don’t know	1 (5)	3 (9)	4 (7)

^a^Since decimals were omitted, the sum is not necessarily 100 (%) for each parameter

**Fig. 3:**
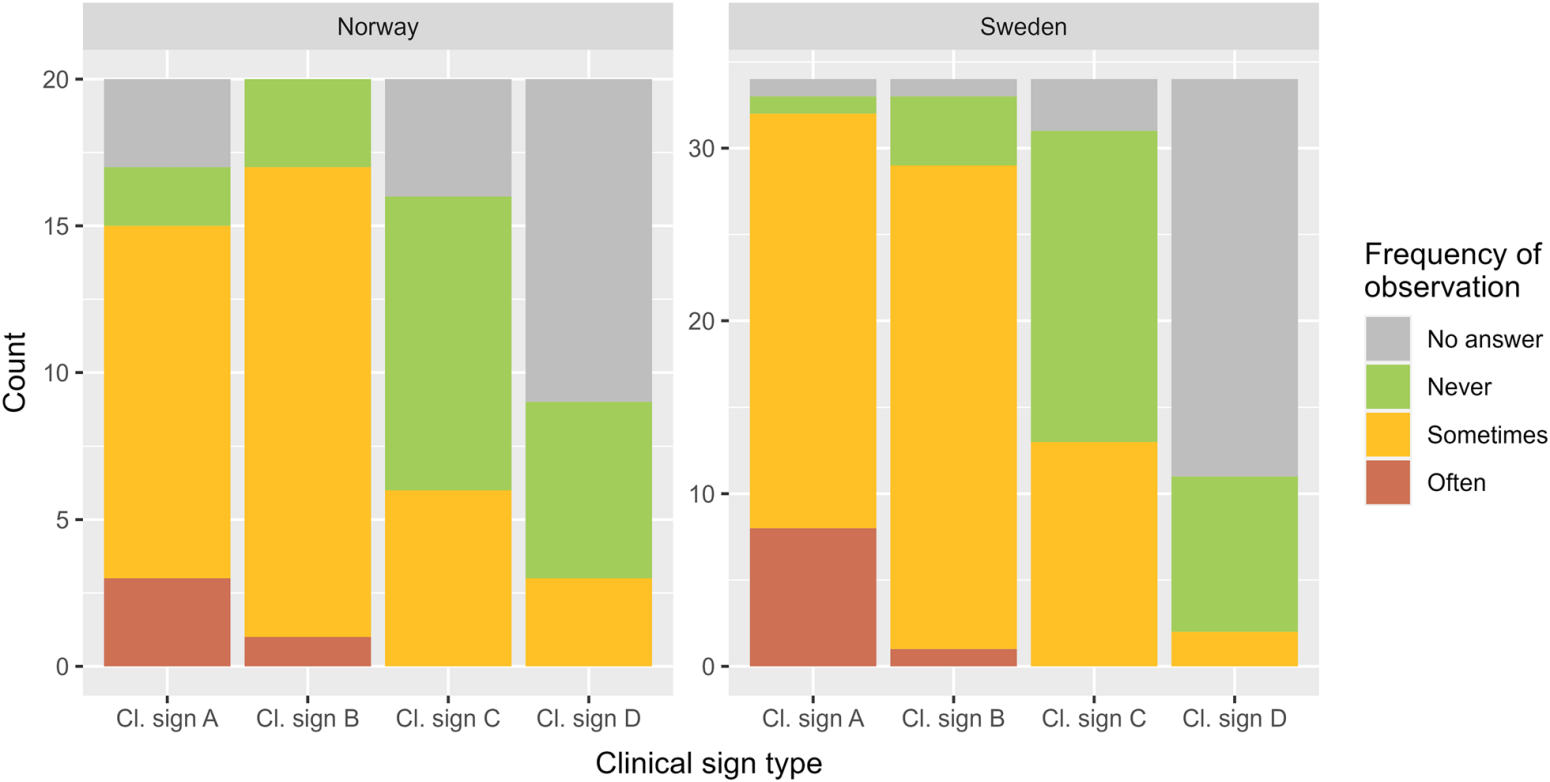
Frequency of clinical signs associated with different stages of infectious keratoconjunctivitis identified by 54 respondents, 20 from Norway respective 34 from Sweden, in the questionnaire survey regarding health and supplementary feeding of semi-domesticated reindeer. Clinical sign A represents increased lacrimation (epiphora), causing a “wet cheek” (mild stage); B represents conjuctivitis and bluish/whitish discoloration of the cornea due to edema (moderately severe stage); and C represents periorbital edema and swelling (chemosis) and shedding of pus (very severe stage). D represents clinical signs’other’ than A–C

The most observed clinical signs in both countries, observed sometimes or often, were epiphora/increased lacrimation causing a “wet cheek” (Fig. 1a) and conjunctivitis and bluish/whitish discoloration of the cornea due to oedema (Figs. 1b, 3). Of the 54 respondents that had observed IKC or other clinical signs in the past 10 years, 47 (87%) observed clinical sign A sometimes or often, with 46 reporting clinical sign B (85%). The least frequently observed clinical signs were C (periorbital oedema and swelling (chemosis), and shedding of pus, Fig. 1c), and clinical sign D, representing clinical signs ‘other’ than A–C (Fig. 3). Other clinical signs mentioned in the comments were ruptured eyes, purulent secretions and white spots on corneas.

### Observed clinical signs related to season and age

**Fig. 4:**
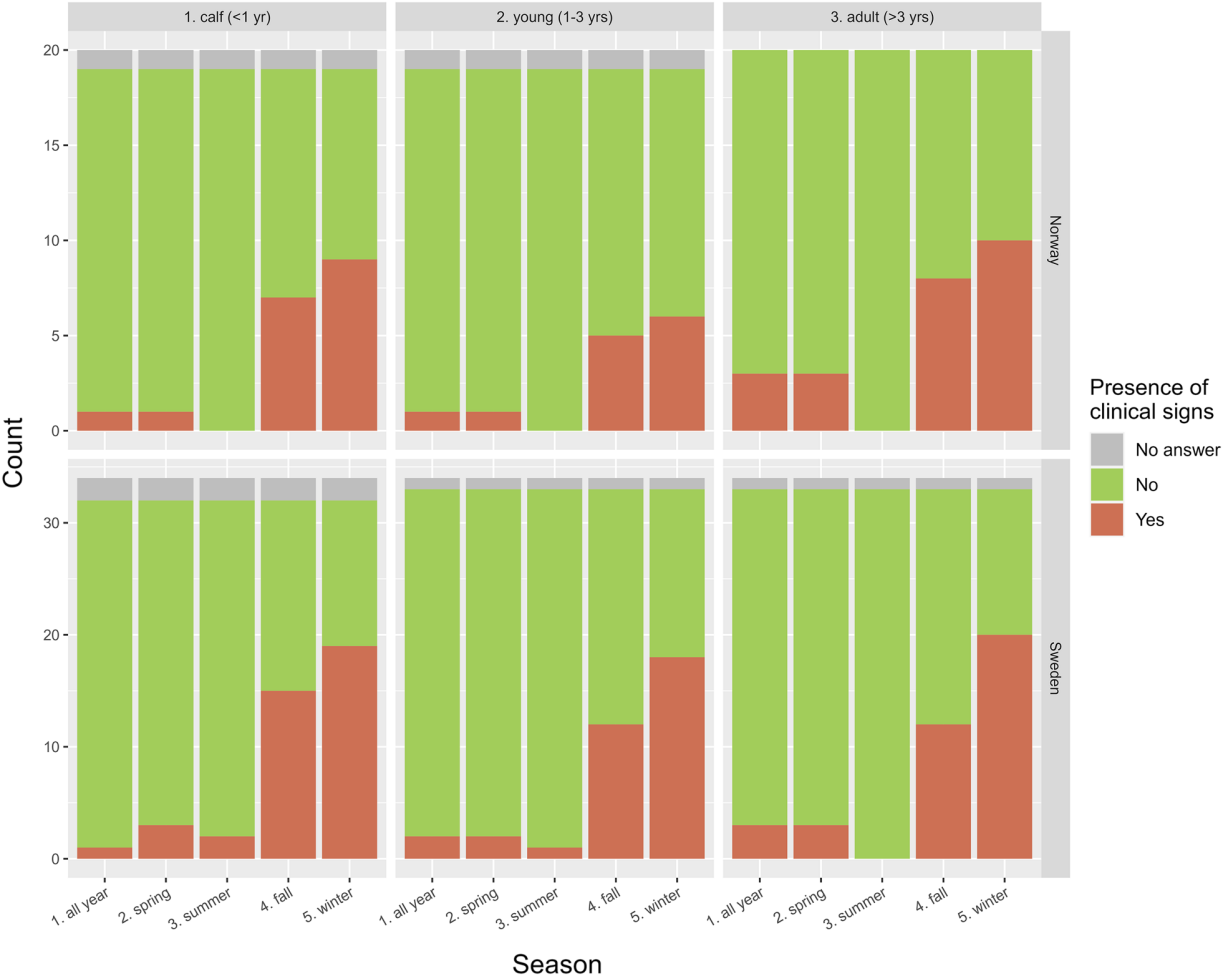
Reported observations of infectious keratoconjunctivitis over the four seasons of a year, in age categories: calves (< 1 year), young reindeer (1–3 years), and adults (> 3 years), from 54 herders surveyed in the questionnaire regarding health and supplementary feeding of semi-domesticated reindeer, 20 respondents from Norway and 34 from Sweden

Clinical signs of IKC were mainly observed during the autumn and winter season for all three age categories: calf, young and adult (Fig. 4). A minority of respondents reported IKC all year round or in spring. In Sweden, calves and young reindeer were the only age categories observed with clinical signs during the summer. Comments to this set of questions reported that all three stages of eye disease were observed, the most common being increased lacrimation in calves during autumn and winter. Respondents also stated that dust from the feed was a cause of IKC, and finally, that clinical signs mostly appeared in calves and during supplementary feeding.

Some direct quotes (opinions) from free text comments (voluntary to fill out) by respondents in the questionnaire, related to IKC were:

“*I often observe wet cheeks all year round.*”

“*We rarely observe eye diseases but have experienced all clinical signs given in the questionnaire, most common is tear flow from calves during autumn and winter.*”

“*Mostly observed in calves during supplementary feeding.*”

“*It can spread rapidly among reindeer whatever their condition.*”

### Supplementary feeding

**Fig. 5:**
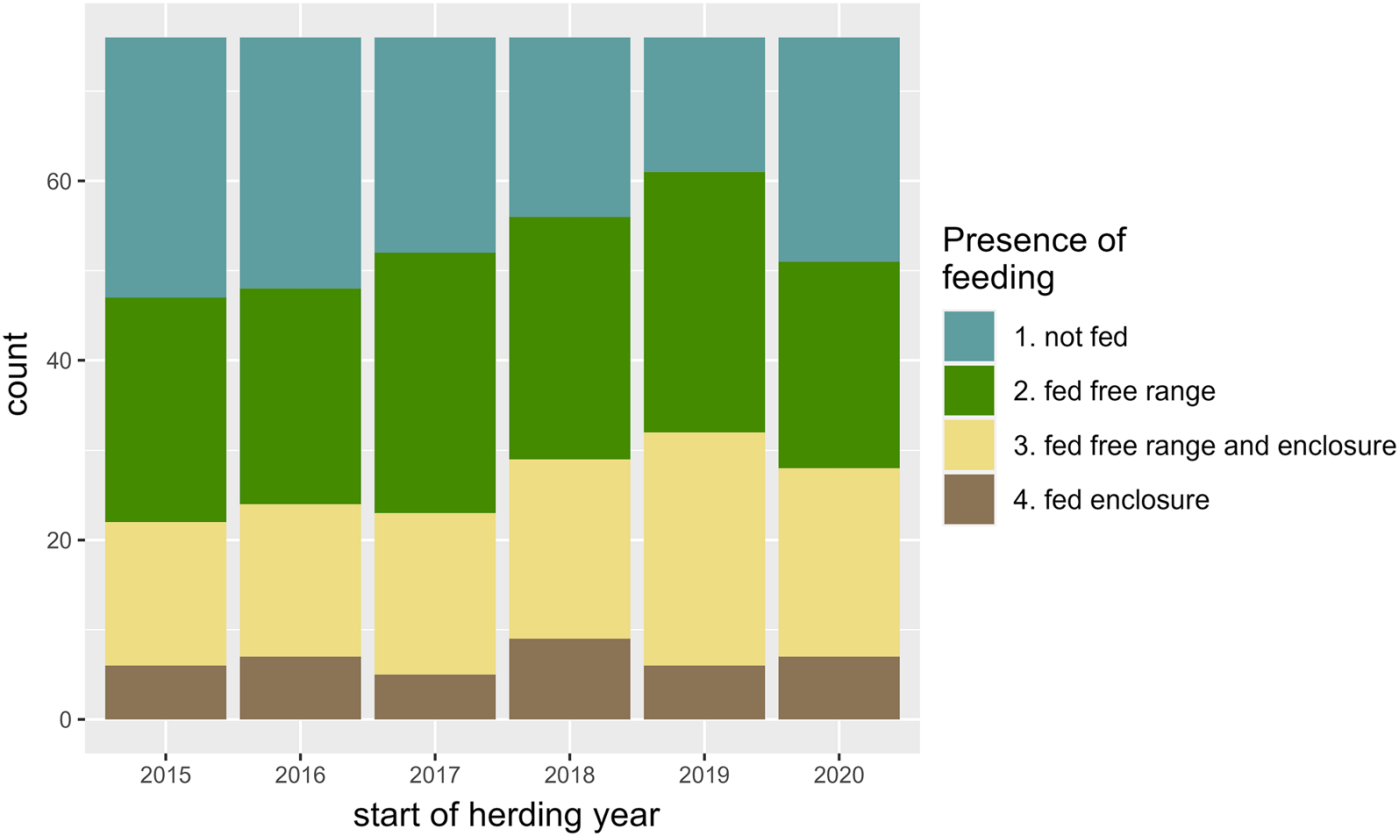
Distribution of feeding practices over six herding years, from 2015/2016 to 2020/2021, based on answers from herders who responded to the questionnaire survey regarding the health and supplementary feeding of reindeer in Norway and Sweden. Sixty-six respondents, 27 from Norway and 39 from Sweden, had performed supplementary feeding at some time over the past 5 years, whereas ten respondents answered that their animals were free-range with no supplementary feed at all

In total, 66 (87%) of the respondents had performed supplementary feeding from time to time over the past 5 years, 27 (82%) of the Norwegian respondents, and 39 (91%) of the Swedish respondents. In general, over the past five herding years, free-range feeding was most common, followed by a mix of free-range feeding and in enclosure feeding for a single herd during the herding year. The least common was feeding in enclosures only (Fig. 5). The herding year in which most of the respondents performed feeding was 2019/2020, in which 61 (80%) out of 76 respondents fed their reindeer (Fig. 5).

In general, larger groups of reindeer (≥ 300) were commonly free-range fed, while smaller groups of reindeer (< 300) were fed in enclosures more often, only investigated for the herding year 2019/2020. In addition, it was more common (n = 15; 35%) to only feed calves in enclosures in Sweden, compared to feeding the whole group (n = 3; 7%). This was in contrast to Norway where it was unusual to feed calves as well as the whole group in enclosures (Table 3). None of the respondents specified that they had exclusively fed reindeer over 1 year of age. Additional comments mentioned that certain groups of reindeer were fed each year, including calves, weak animals, animals selected for slaughter, reindeer involved in tourism and tame reindeer, while the rest of the group was not fed.

**Table 3:** Distribution of the number of reindeer and the animal category (e.g., calves or whole group) fed in an enclosure or free-range fed, investigated for the herding year 2019/2020. Presented per country out of the 76 reindeer owners from Norway (n=33) and Sweden (n=43) who responded to the questionnaire survey regarding health and supplementary feeding of reindeer

Question	In enclosures			Free-range		
	Norway n (%^a^)	Sweden n (%^a^)	Total n (%^a^)	Norway n (%^a^)	Sweden n (%^a^)	Total n (%^a^)
Number of animals fed						
No answer	1 (3)	5 (12)	6 (8)	1 (3)	5 (12)	6 (8)
None	22 (67)	16 (37)	38 (50)	7 (21)	11 (26)	18 (24)
< 300	7 (21)	10 (23)	17 (22)	3 (9)	5 (12)	8 (11)
≥ 300	3 (9)	12 (28)	15 (20)	22 (67)	22 (51)	44 (58)
Animal category						
No answer	2 (6)	5 (12)	7 (9)	2 (6)	5 (12)	7 (9)
None	24 (73)	20 (47)	44 (58)	7 (21)	12 (28)	19 (25)
Calves only	3 (9)	15 (35)	18 (24)	0 (0)	0 (0)	0 (0)
Whole group	4 (12)	3 (7)	7 (9)	24 (73)	26 (60)	50 (66)

^a^Decimals are omitted and therefore the sum is not 100 (%) for each column

Some direct quotes (opinions) from free text comments (voluntary to fill out) by respondents in the questionnaire, related to supplementary feeding were:

“*I feed a few reindeer every winter.*”

“*The majority are free-range fed.*”

“*Some winters, we separate the calves to feed them in enclosures, and the rest of the herd are free-range fed.*”

### Outbreak of infectious keratoconjunctivitis per herding year and number of affected reindeer

**Fig. 6:**
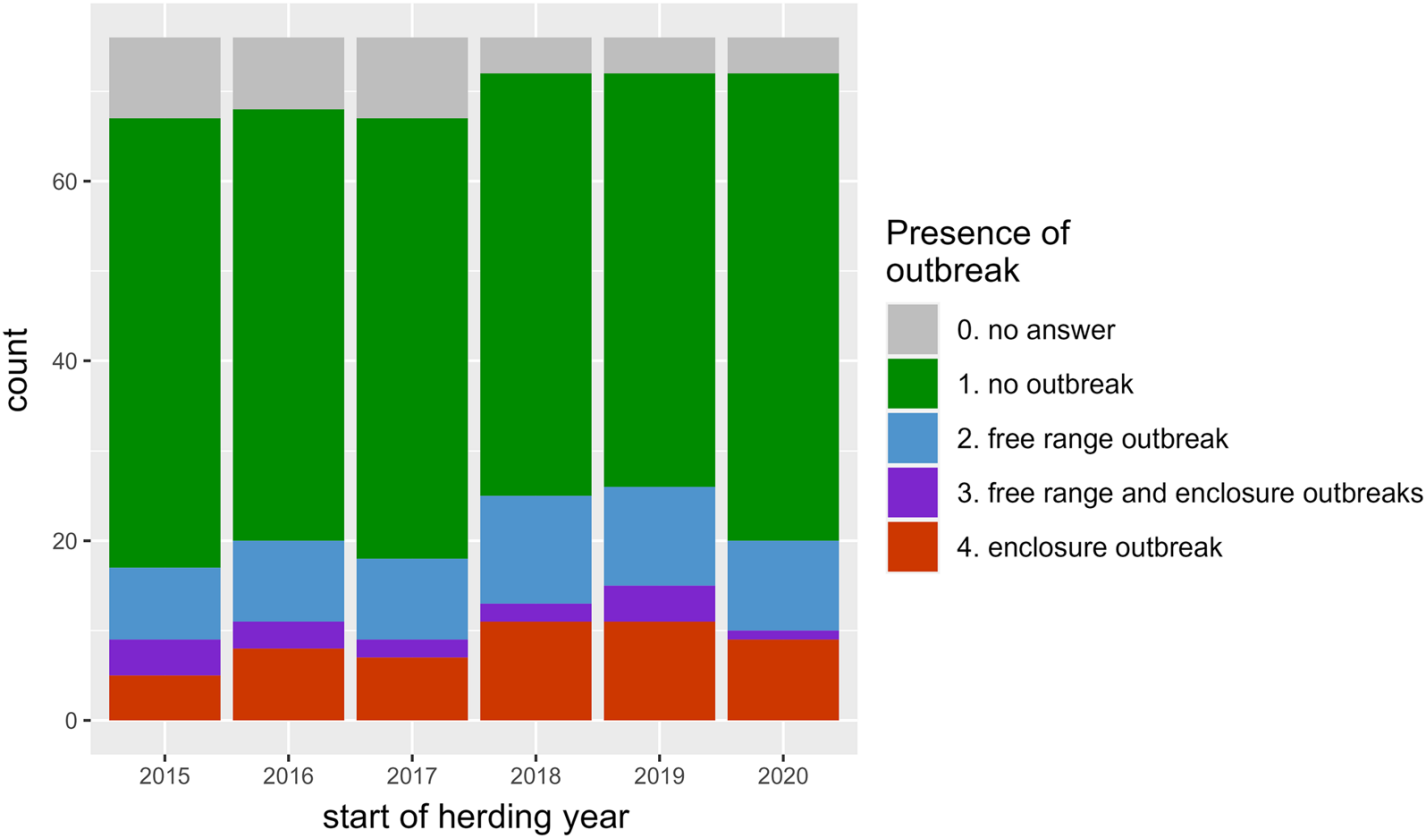
Observations of outbreaks of infectious keratoconjunctivitis at herd level, distributed as free-range and/or enclosure, independent of whether the reindeer were fed or not, over six herding years, from 2015/2016 until 2020/2021. The figure is based on answers from 76 herders who responded to the questionnaire survey regarding the health and supplementary feeding of reindeer in Norway and Sweden

For each herding year in the period 2015/2016–2020/2021, the majority of the respondents had not reported an outbreak of IKC (Fig. 6). Outbreaks of IKC were most commonly reported during the herding year 2019/2020, when 20 (26%) of the 76 respondents reported an outbreak. During the same year, there was no difference in the number of reported outbreaks among herds in enclosures compared to free-range herds, 11 (15%) responders reported outbreaks under one of the two conditions, while just four respondents (6%) reported outbreaks in both enclosure and free-range (Fig. 6). In general, the same pattern was repeated each herding year; outbreaks were most commonly observed in enclosures or in free-range herds and were less commonly reported in both enclosure and free-range, independent of whether the reindeer were fed or not. The proportion of affected reindeer varied greatly, from 0.2 to 100% of reindeer being affected in one group. In general, calves (< 1 year) and young reindeer (< 3 years) were reported to be affected in greater numbers, spanning from 0.2 to 100%, compared to adult reindeer (3 years or more), spanning from 0 to 20%. Since information on the age composition of the total herd from which the affected reindeer were part was missing, the true proportion of affected calves/young and adult reindeer could not be calculated. However, herders with herds up to around 100 animals tended to report a higher proportion of affected reindeer (20–100%) compared to herders with larger herd sizes (around 2000), who reported a lower proportion of affected reindeer (0.1–1%). Several of the respondents commented that they had seen individual animals with IKC but had not experienced an outbreak.

Some direct quotes (opinions) from free text comments (voluntary to fill out) by respondents in the questionnaire, related to outbreaks of IKC and season were:

“*I have observed reindeer with clinical signs but not experienced an outbreak during 2020/2021, the disease does not seem as contagious this year.*”

“*It has been more or less common for one year depending on where the reindeer were located during the autumn.*”

“*We usually observe a few reindeer with clinical signs of different severity, it is easier to observe the disease during the winter.*”

### Relationship between feeding and outbreak of infectious keratoconjunctivitis

When considering the year-based questions, there were 456 herd-year observations. However, due to non-answers from some herders to the year-specific herd question, 418 complete observations were available for regression modelling.

Univariable mixed effect regression models showed no evidence of an association between feeding overall and the presence of outbreaks in a herd. However, when separating feeding by location, e.g., free-range versus enclosure, we observed that feeding in enclosures was significantly associated with higher odds of IKC outbreak (Odds Ratio = 11.59, *P* = 0.011). Free-range feeding had a negative, but non-significant, relationship with the appearance of an IKC outbreak (Table 4).

**Table 4:** Results from a univariable mixed-effect logistic regression, where the observed presence of an outbreak of infectious keratoconjunctivitis in a herd for a given herding year was the outcome, with each observation under analysis being a herding year per herd (number of herd-years). Based on the 76 herders surveyed in the questionnaire regarding health and supplementary feeding of reindeer, 33 respondents from Norway and 43 from Sweden

Variable	Category	Numbers of herd-years	Numbers of herd-years with outbreak	Proportion	OR univariate	95% CI	*P*-value
Feeding overall	No	134	33	0.25	Reference		
	Yes	284	93	0.33	1.30	0.15–11.58	0.82
Feeding in enclosure	No	276	68	0.25	Reference		
	Yes	142	58	0.41	11.59	1.75–77.01	0.01
Free-range feeding	No	167	43	0.26	Reference		
	Yes	251	83	0.33	0.29	0.05–1.72	0.17
Herd size	< 500	134	40	0.30	Reference		
	500–999	83	29	0.35	1.54	0.02–123.80	0.85
	1000–1999	82	16	0.20	0.88	0.01–102.49	0.96
	> 1999	119	41	0.35	1.14	0.02–71.51	0.95
Regions	Norrbotten	129	45	0.35	Reference		
	Västerbotten	56	15	0.27	1.33	0.01–211.03	0.91
	Dalarna/Jämtland	36	6	0.17	0.21	0.00–305.97	0.68
	Finmark	102	30	0.29	0.50	0.01–38.98	0.76
	Nordland/Troms	59	24	0.41	1.23	0.01–213.52	0.93
	Trondelag/Hedmark	36	6	0.17	0.21	0.00–305.94	0.68
Country	Norway	197	60	0.31	Reference		
	Sweden	221	66	0.30	1.48	0.06–36.36	0.81
Herding year	2015–2016	67	17	0.25	Reference		
	2016–2017	68	20	0.29	3.21	0.42–24.73	0.26
	2017–2018	67	18	0.27	1.36	0.17–11.02	0.77
	2018–2019	72	25	0.35	6.83	0.89–52.29	0.06
	2019–2020	72	26	0.36	10.14	1.29–79.90	0.03
	2020–2021	72	20	0.28	0.81	0.11–6.15	0.84

OR, odds ratio; CI, confidence interval

The multivariable model selection led to the inclusion of the location specific feeding in enclosures variable described above as well as the herding year. The association with feeding practice was similar when seen in the multivariable model, with an OR of 15.20 for feeding in enclosures (*P* = 0.009). The herding years 2018–2019 and 2019–2020 were associated with a higher likelihood of an outbreak of IKC compared to the reference year 2015–2016, with 2019–2020 having the highest likelihood (OR = 13.04, *P* = 0.027). The other 3 years (2016–2017, 2017–2018, and 2020–2021) were not significantly different from 2015–2016 (Table 5).

**Table 5:** Results from multivariable model selection, where the observed presence of an outbreak of infectious keratoconjunctivitis in a herd was the outcome, based on 76 herders surveyed in the questionnaire regarding health and supplementary feeding of reindeer, 33 respondents from Norway and 43 from Sweden

Variable	Category	OR multivariate	95% CI	*P*-value
Feeding in enclosure	No	Reference		
	Yes	15.20	1.95–118.53	0.01
Herding year	2015–2016	Reference		
	2016–2017	3.65	0.41–32.78	0.25
	2017–2018	1.17	0.13–10.64	0.89
	2018–2019	7.40	0.81–67.70	0.08
	2019–2020	13.04	1.34–127.37	0.03
	2020–2021	0.80	0.09–7.28	0.84

OR, odds ratio; CI, confidence interval

Forcing the free-range feeding variable into the multivariate model, led to similar results for the prior two variables and a negative borderline significant association between free-range feeding and the presence of an IKC outbreak (OR = 0.17, *P* = 0.0996).

### Changes in the occurrence of infectious keratoconjunctivitis, management, and access to veterinary assistance

Twenty-three (43%) of the respondents, nine from Norway and 14 from Sweden, stated that they had not observed any changes in the occurrence of IKC over the past 5 years. Six herders (11%) answered that the disease had decreased, and 12 (22%) stating that they did not know. Answers were distributed equally between Norwegian and Swedish respondents. Thirteen (24%) respondents, one from Norway and 12 from Sweden, stated that the number of IKC cases had increased. Comments on why IKC had increased included an increase in supplementary feeding, stress, and climate change, which had caused warmer and wetter weather conditions.

Some direct quotes (opinions) from free text comments (voluntary to fill out) as to why IKC had increased:

“*Limited access to natural pastures has led to feeding in enclosures the last couple of years.*”

“*Dust from the feed and close contact between the reindeer.*”

“*Warmer and wetter weather.*”

Comments on why IKC had decreased focused on the use of better enclosures, better monitoring and awareness, and the reduction of stress in their reindeer.

Some direct quotes (opinions) from free text comments (voluntary to fill out) as to why IKC had decreased:

“*We are more careful and aware, sick reindeer are isolated and we change clothes between handling sick and healthy reindeer.*”

“*It depends on how much stress there is, from mosquitos and from other sources, we also perform free-range feeding.*”

**Fig. 7:**
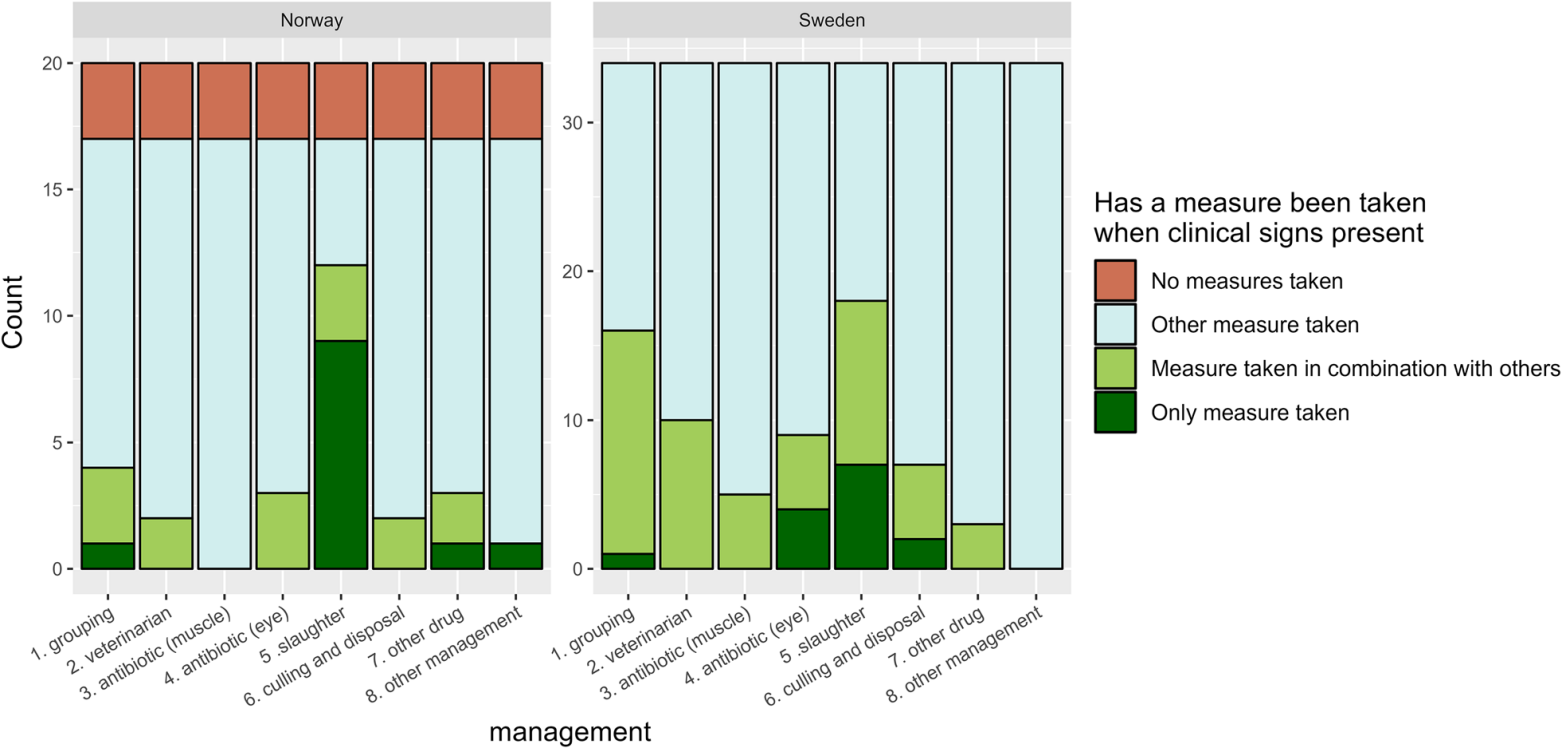
Reported measures when observations of clinical signs of infectious keratoconjunctivitis were last made, from the 54 herders that had observed clinical signs of IKC over the past 10 years, surveyed in the questionnaire regarding health and supplementary feeding of semi-domesticated reindeer, with 20 respondents from Norway and 34 from Sweden

Forty-one (76%) of the respondents, 13 from Norway and 28 from Sweden, usually acted when IKC was observed in their reindeer. Different measures were performed when clinical signs of IKC were last observed by the herders. Slaughter was the most common measure (n = 30; 56%), followed by grouping and isolation of affected reindeer (n = 20; 37%), for both countries (Fig. 7).

Three herders commented in the comment field that they performed eye washing. The majority of the respondents (n = 37, 70%) had not heard about any traditional treatments other than the slaughter of calves, eye washing, or other antimicrobial or antibiotic treatment administrated locally in the affected eye. Three herders also mentioned snuff in the eye as a treatment performed in the past.

Most of the respondents (n = 39, 74%), distributed equally between Norway and Sweden, did not think or did not know whether IKC had brought any economic consequences to them. Only 14 herders (26%), three from Norway and 11 from Sweden, stated that IKC had brought economic consequences. Thirty-eight of the respondents (14 from Norway and 24 from Sweden, a total 58% of the 76 respondents) were able to get the help they needed from a veterinarian. The other 42% answered that they could not get the help they needed from a veterinarian. The answers were distributed equally between Norwegian and Swedish respondents. The lack of knowledge of reindeer diseases among veterinarians was a frequently stated cause in the comment field.

Some direct quotes (opinions) from free text comments (voluntary to fill out) by respondents in the questionnaire, related to access to veterinary assistance were:

“*It is difficult to get hold of a veterinarian with knowledge of reindeer.*”

“*Often the veterinarian does not know what to do, nor can they help to determine the cause of the sick reindeer.*”

“*I think I can get help if needed, but they are too far away and too expensive.*”

“*Lack of availability and knowledge.*”

Finally, the Sámi traditional names for IKC mentioned in the comments were: “Golgii čalbmi” (tearflow), “Deiggan” (white eye), “Čalbme vihkki” (eye disease), and in Swedish “Blåöga” (blue eye).

## Discussion

This study presents an overview of the current presence of IKC in Norway and Sweden, as reported by the reindeer herders, and confirms that IKC is frequently or sporadically observed. We also found a significant association between outbreaks of IKC and feeding when performed in an enclosure. However, free-range feeding seemed to have a potentially protective influence on IKC.

### Presence and clinical signs of infectious keratoconjunctivitis

Our study revealed that a high proportion (n = 54, 71%) of herders had observed clinical signs of IKC, being mostly mild to moderately severe (images a and b in Fig. 1). This finding is in line with the previous survey performed in 2011 [34], where 55% of the herds reported signs of IKC. Clinical signs like “wet cheeks” are easily seen from a distance on freely grazing animals. This may represent a bias and reason why this is most often observed and reported in our study. Moreover, the reindeer herders might have learnt that this is an early clinical sign of IKC that needs further attention. Autumn and winter were the two seasons when most cases of IKC were reported. The animals are exposed to stress and are kept at a higher density during these seasons, which could potentially initiate IKC and its transmission. In the questionnaire survey performed in 2011 [34], the main seasonal appearance of the disease was from September to November, corresponding to autumn in our study. However, the potential shift towards IKC being mostly observed in winter may be associated with the reported increase in emergency feeding practices to prevent starvation which have taken place in recent years in both countries due to unfavorable weather conditions, as well as loss of pastureland because of competing land use [6, 7]. Yet, another potential explanation to differences in the seasonal appearance of IKC would be the different pathogens which could be involved, some of which may be more suited to different settings, herds, or seasons. For example, *Listeria monocytogenes* has been reported in outbreaks as a causative agent of IKC in Finland and are known to be associated with feeding silage [31]. In addition, co-infections can appear, with CvHV2 able to facilitate secondary infections caused by other pathogens, e.g., *Fusobacterium necrophorum*, an obligate anaerobic bacterium causing the disease necrobacillosis, as well as orf virus (ORFV; genus *Parapoxvirus*, family *Poxviridae*), which causes contagious ecthyma [32]. In a recent study, all three pathogens were identified during the same IKC outbreak, indicating that they may have interacted with each other or caused co-infections [31]. A novel gammaherpes virus (*Rangiferine gammaherpesvirus 1*) has recently been identified in reindeer in Norway, and a high seroprevalence of gammaherpes virus was found in reindeer in Finland, with unknown pathological potential in either country [25, 33]. Viruses from this group could play a part in the pathogenesis of IKC. Microbiological studies are, however, warranted to further clarify the pathological impact and potential associations between gammaherpes viruses and ocular disease in reindeer. One possible constraint is the responders’ inability to distinguish between different causes, like trauma, insects or microbial agents leading to clinical signs similar to IKC. However non-infectious causes should affect single cases rather than outbreaks of IKC.

### Supplementary feeding

Supplementary feeding was commonly performed by the herders, where 66 (87%) had fed their reindeer at sometime during the past 5 years, most often free-range feeding. Our results significantly point to the herding year of 2019/2020 as having the highest number of reports of herds with outbreak of IKC and being the time when most of the herders (80%) had performed supplementary feeding. This is in line with several sources reporting this winter as catastrophic because of the lack of availability of winter pastures, causing many herders to apply for economical support because of costs related to supplementary feeding [6]. Our results are in contrast with the previous survey in 2011 [34], where environmental factors, e.g., supplementary feeding, were not associated with clinical signs of IKC, suggesting there has been an increase of outbreaks of IKC associated with feeding over the last decade. However, in the previous study, the distribution of respondents between Norway and Sweden was different, with only 15 respondents from Sweden (24%), limiting the comparability of the results. The majority of respondents in our survey stated that they had not observed any changes in the occurrence of the disease. In Sweden, however, the number of herders reporting an increase was almost as many as reporting a decrease, whereas this was not the case in Norway. This could potentially mean that there are differences between the countries, and that IKC might have increased in Sweden. One explanation for this could be that herders in Sweden more commonly fed calves in enclosures compared to Norway, where feeding in enclosures was less common overall (Table 3).

The present study showed a trend of a higher proportion of calves reported with clinical signs of IKC, than adults. Also, herders with smaller groups of reindeer (≤ 100) seemed to be reporting a higher proportion of affected animals than herders with larger herds. In addition, our results revealed that smaller groups of reindeer (< 300) were more often fed in enclosures compared to larger (> 300) groups (Table 3). Several herders, however, commented that they had observed isolated cases of IKC but had not experienced an outbreak. This could mean that the herder’s ability to detect IKC has improved rather than there has been an increase in outbreaks of IKC.

### Association between feeding and outbreak of infectious keratoconjunctivitis

Another possible explanation for why feeding in enclosures was associated with reports of outbreak of IKC in a herd could be that calves and young reindeer are more sensitive to stress and susceptible to infection and are more likely to be fed whole rations in enclosures compared to adult reindeer and the whole winter group. This is in line with previous studies that point to management factors, increased animal density, stress, and challenging hygienic conditions as risk factors for transmission and outbreak of IKC, primarily affecting calves and young reindeer [21–23]. The separation of mother and calf further adds to stress that could lead to reactivation of CvHV2 [23, 24]. Hence, we find it likely that the potential increase in the animals’ stress levels, combined with the increased contact between infected and immunologically naïve animals, could have a greater impact on the occurrence of IKC than supplementary feeding itself. The density of animals in an enclosure is higher compared to free-range, despite the fact that herders reported a smaller group size in total in enclosures. While for free-range animals, it is easier to keep better hygienic conditions, with a regular change of feeding site, access to clean snow or water, and where natural pasture is not restricted to the same extent as in an enclosure [6, 14]. However, in this study, questions about the enclosures such as animal density, size, and topography were not included, which are factors that could play a role in the development of infectious diseases like IKC. This needs to be further studied, especially when it is getting more challenging to keeping reindeer in enclosures due to climatic changes [1].

In our study, dust from the feed was mentioned in the comments as a contributing factor for IKC. Early studies suggest that both summer and autumn, as well as spring, were seasons where clinical signs of IKC could be observed. This was reported mainly in forest reindeer because of corralling in dusty environments and heavy loads of insects leading to foreign bodies and corneal lesions, and eyelid irritation (caused by insect bites) as initial causes, paving the way for secondary infections, leading to IKC. The herding conditions for mountain reindeer differed and could be used as an argument as to why they seemed to have fewer cases of infected eyes in this herding district type [18, 19]. However, another study reported an outbreak of IKC in calves supplementary fed during winter, with management factors causing stress and feed particles from pellets being suggested as the main contributors leading to secondary infections and, consequently, IKC [20]. The present research still addresses the disease as multifactorial, where stress (associated with herding, gathering, transport, and translocation to new environments), environmental conditions and management factors are associated with IKC. Dust and other sorts of trauma can harm the eye without being the direct cause of IKC. In addition, CvHV2 has recently been established as a primary, causative agent, while other agents, like *Chlamydia*, and their role in the pathogenesis of IKC need to be further clarified [21–23].

Unexpectedly, free-range feeding had a non-significant, but negative relationship with the presence of an outbreak of IKC. Thus, feeding on free-range could potentially be protective against the presence of an outbreak of IKC in a herd. Our results revealed that larger groups of reindeer (> 300) and the whole winter group were most often free-range fed (Table 3). Access to natural pastureland, better hygienic condition due to larger areas to utilize, and no additional stress because of handling and separation of female and calf when free-ranging could be potentially important prophylactic factors against an outbreak of IKC. In addition, nutritional status and general condition are crucial for the survival of the animals. Supplementary feeding, conducted in the right manner, such as with a gradual introduction to the diet under good hygienic conditions, and with high quality feed suited to reindeer, contributes to increased body weight and general health condition. Consequently, better resilience against stress and diseases, increased reproduction success, and a decrease in calf mortality is expected and has been documented [10, 35].

However, it should be noted that this information regarding season and location in association with IKC could be biased, since most reindeer are handled and inspected at close range due to management practices during winter and autumn. Reindeer kept in enclosures are also observed more closely, thus it is easier to detect signs of IKC compared to free-range reindeer, which limits the interpretation of our results. Future studies investigating the relationship between the presence of outbreaks in an enclosure compared to free-range outbreaks in more depth and on an individual animal-level are therefore warranted. This could be investigated by performing in-depth interviews specifically addressing these issues.

### Management of infectious keratoconjunctivitis

Forty-one (76%) out of the 54 respondents who had experienced IKC usually took measures to address it, with the most commonly practiced action being slaughter, but antibiotic treatment and contact with a veterinarian was also reported. Furthermore, herders reported that reindeer with IKC were isolated from the rest of the herd (Fig. 7). This is in contrast to the results in the previous study by Tryland et al. [34], where none of the herders isolated affected reindeer. This change in management might be due to increased awareness and knowledge of the disease. We also noted that several herders reported a general lack of access to veterinarians with thorough knowledge of reindeer diseases as a reason for not getting adequate help from a veterinarian. Although an increase was observed, from 7 (12%) to 12 (22%) of the respondents in the previous survey from 2011 and in our survey, respectively, it may still be regarded as uncommon to contact a veterinarian when IKC appears. Antibiotic treatment administered locally was reported to be used to a higher extent than systemic administered antibiotics. There are no approved eye ointments for reindeer, so all use is off-label [36, 37].

Most of the respondents from Norway were from Finnmark (51%) but the reported herd size in Vest-Finnmark was high, with herd sizes of over 2000 reindeer. In Norway, 70% of all semi-domesticated reindeer and 75% of all reindeer owners are located in Finnmark (Vest- and Øst-Finnmark included), and the average number of reindeer is about 65 per owner. However, 80% of the owners in this area have less than 50 reindeer, and the highest proportion of small herd size is in Vest-Finnmark. In addition, the reindeer number of each reindeer herding group (siida-share) varies within each region [8]. This might influence our results and the respondents might be less representative of that specific region. Most of the respondents from Sweden were from Norrbotten (56%), where the stated herd size varied significantly and may not be representative of general reindeer owners in the region. There are more owners and reindeer in Norrbotten, mostly made up of small enterprises with fewer reindeer per herder than in Västerbotten, Jämtland and Dalarna [8].

We know that there are great variations between regions within each country with regards to geography, herding area, herd size, number of reindeer owners and herders, other land users, and density of different predators, all affecting profitability and production. Therefore, we investigated differences between regions in each country when possible. There are also differences in reindeer herding between Norway and Sweden (e.g., differences in governance, economic support system, geography, herding area, predator management policies, legislation, access to veterinarians, and management practices) that could influence our results, considering our relatively few respondents. However, there are also similarities, for example, some mountain districts in Sweden migrating to Norway for summer pasture. In addition, reindeer pastoralism in Fennoscandia faces the same kind of challenges due to climate change and competing land use in the reindeer herding area [8]. Our findings showed no association between the appearance of IKC and herd size, region, or country.

### Limitations

Besides the potentially questionable geographical representativity of our respondents, the population of reindeer herds is relatively small in both countries, and of this, we only got an approximately 5% response rate in each country. Since we found an equal distribution of data between the two countries, we decided to present the data as one population (Norway and Sweden together), and most results are presented using a descriptive approach. The relatively small number of respondents could be the reason why this study potentially lacked the power to reveal certain associations, such as the non-significant negative relationship between outbreaks of IKC and supplementary free-range feeding. Another possible challenge may be recalling bias, as we go back 5–10 years for many of our questions. In addition, potential selection bias should also be considered, since herders more experienced with IKC and/or supplementary feeding might have been more motivated to respond to our survey. This could lead to several biases in the data. For example, herders responding to the questionnaire could have observed IKC more often than the general herder and therefore made more measures to stop the transmission of the disease. In addition, the respondents might have recognized early clinical signs more than general herders or may have had more experience in feeding their animals. Also, clinical signs of IKC are not specific for infectious agents and could also be caused by insects and trauma. Therefore, we cannot rule out that the reported cases of IKC in our study might be of a non-infectious origin. However, the questions in the questionnaire pointed out that we are interested in outbreaks, not single cases. Because the study was retrospective and covered several years, we could not ask for detailed information about where and when feeding was practiced in relation to outbreaks of IKC, and there was a lack of information at the animal level. Therefore, we could only investigate associations between outbreaks and feeding on a herd level and not on an individual level. Thus, we may have missed information on whether the same animals that have experienced an outbreak, or had been observed with IKC, had also been supplementary fed or not, and in what environmental conditions. Follow up studies, such as in-depth interviews, to investigate this closer and, if possible, to do so at an individual level would further clarify the relationship between IKC and feeding in enclosures compared to free-range.

## Conclusions

Clinical signs of IKC were commonly observed at a herd level, and the main seasonal appearance of IKC were during the autumn and winter. Mild to moderate clinical signs were most commonly observed, and slaughter, followed by isolation of affected animals from the herd, was the most common measure taken by the herders. This could indicate an increased awareness and knowledge of the disease today compared to a decade ago. However, herders were reluctant to contact a veterinarian and a common reason mentioned was that veterinarians had restricted knowledge about reindeer diseases. In addition, feeding in enclosures, which is performed mainly during the winter season, was associated with an outbreak of IKC in a herd, whereas free-range feeding could potentially be protective. The known catastrophic herding year 2019/2020, which had inaccessible winter pastures, was significantly associated with the presence of an outbreak of IKC in a herd, as well as being the herding year where most herders had performed supplementary feeding. Results imply that infectious diseases like IKC are associated with the feeding situations, affecting calves especially. In-depth studies focusing on the animal level could further clarify these associations.

## Supplementary Information

**Additional file 1**: The Questionnaire in Swedish.

**Additional file 2**: The Questionnaire in Norwegian.

**Additional file 3**: The Questionnaire in North Sámi.

**Additional file 4**: Specified herd size per reindeer herding region in each country from 76 respondents, 33 from Norway and 43 from Sweden.

## Data Availability

The datasets used and/or analyzed during the current study are available from the corresponding author on reasonable request. The questionnaire, for Swedish, Norwegian, and Northern Sámi, used in the study is to be found in additional files (Additional files 1, 2, 3).

## Declarations

### Prior publication

Data has not been published previously.
