# Supplementary material for: Infectious keratoconjunctivitis in semi-domesticated reindeer (Rangifer tarandus tarandus): a questionnaire-based study among reindeer herders in Norway and Sweden
Source: Acta Vet Scand. 2023 Jul 12;65:34. doi: 10.1186/s13028-023-00694-x (PMC10337086; doi:10.1186/s13028-023-00694-x)
Supplement: Supplementary file 1 — Additional file 1: The Questionnaire in Swedish. [file 13028_2023_694_MOESM1_ESM.pdf]

Additional file 1 The Questionnaire (in Swedish) regarding health and supplementary feeding of semi-domesticated reindeer, given in 2021 and distributed in Norway and Sweden.

Välkommen!

Den här enkäten riktar sig till dig som bedriver renskötsel i Norge eller Sverige, oavsett om du utfodrar eller ej, enkäten förväntas ta upp till 30 minuter att besvara och är uppdelad i flera avsnitt:

1. Frågor om tre smittsamma sjukdomar och om övrig renhälsa.

2. Frågor om utfodring (effekter, rutiner och övrigt).

OBS: Dina SVAR SPARAS EJ om du pausar mitt i enkäten och det går heller EJ ATT GÅ TILLBAKA i enkäten, då försvinner alla svaren och du behöver börja om från början.

Frågor markerade med * är obligatoriska att svara på för att komma vidare.

Om inget annat anges är bilderna i enkäten från SSR:s bildarkiv.

Din identitet kommer att vara dold.

När inget annat anges avser frågorna i enkäten

vinterväderförhållanden under 2019-2020.

Frågorna avser den som svarar på enkäten.

1) * I vilket land bedriver du huvudsakligen renskötsel?

○ Norge

○ Sverige

Denna informationsbox visas endast i läge förhandsgranskningen.

Följande kriterium måste vara uppfyllda för att följande fråga ska visas:

Om frågan I vilket land bedriver du huvudsakligen renskötsel? innehåller något av dessa svarsalternativ

- Sverige

2) * I vilken region bedriver du huvudsakligen renskötsel?

- Dalarna/Jämtland
- Västerbotten
- Norrbotten

Denna informationsbox visas endast i läge förhandsgranskningen.

Följande kriterium måste vara uppfyllda för att följande fråga ska visas:

Om frågan I vilket land bedriver du huvudsakligen renskötsel? innehåller något av dessa svarsalternativ

- Sverige

3) * Ange i vilken typ av sameby du bedriver renskötsel?

- Fjällsameby
- Skogssameby
- Koncessionssameby

Denna informationsbox visas endast i läge förhandsgranskningen.

Följande kriterium måste vara uppfyllda för att följande fråga ska visas:

Om frågan I vilket land bedriver du huvudsakligen renskötsel? innehåller något av dessa svarsalternativ

- Norge

4) * I vilken region bedriver du huvudsakligen renskötsel?

- Øst Finnmark
- Vest Finnmark
- Troms
- Nordland
- Nord-Trøndelag
- Sør-Trøndelag
- Møre- og Romsdal
- Hedmark

Denna informationsbox visas endast i läge förhandsgranskningen.

Följande kriterium måste vara uppfyllda för att följande fråga ska visas:

Om frågan I vilket land bedriver du huvudsakligen renskötsel? innehåller något av dessa svarsalternativ

- Norge

5) * Hur lång erfarenhet har du av att arbeta inom renskötseln?

- Under 5 år
- 5-9 år
- 10-29 år
- Över 30 år

6) Ange kön?

- Kvinna
- Man
- Vill ej ange

7) Ange din ålder?

- Yngre än 20
- 20-39
- 40-59
- över 60

8) * Vad är det ungefärliga antalet djur i din renskötselgrupp (av vinterhjorden), efter årligt slaktuttag?

- Under 50
- 50-99
- 100-249
- 250-499
- 500-999
- 1000-1999
- 2000-2999
- Över 3000

Här följer frågor om tre infektionssjukdomar som kan ha koppling till utfodring.

1. Smittsam ögoninflammation/ögonförändring:

Smittsam ögoninflammation/ögonförändring kan börja med tårflöde som ger blöta kinder. Sjukdomen kan orsakas av både virus och bakterier och är därför smittsam och kan ge upphov till olika ögonförändringar som exempelvis Bild 1. A-C

Bild 1. A-C:

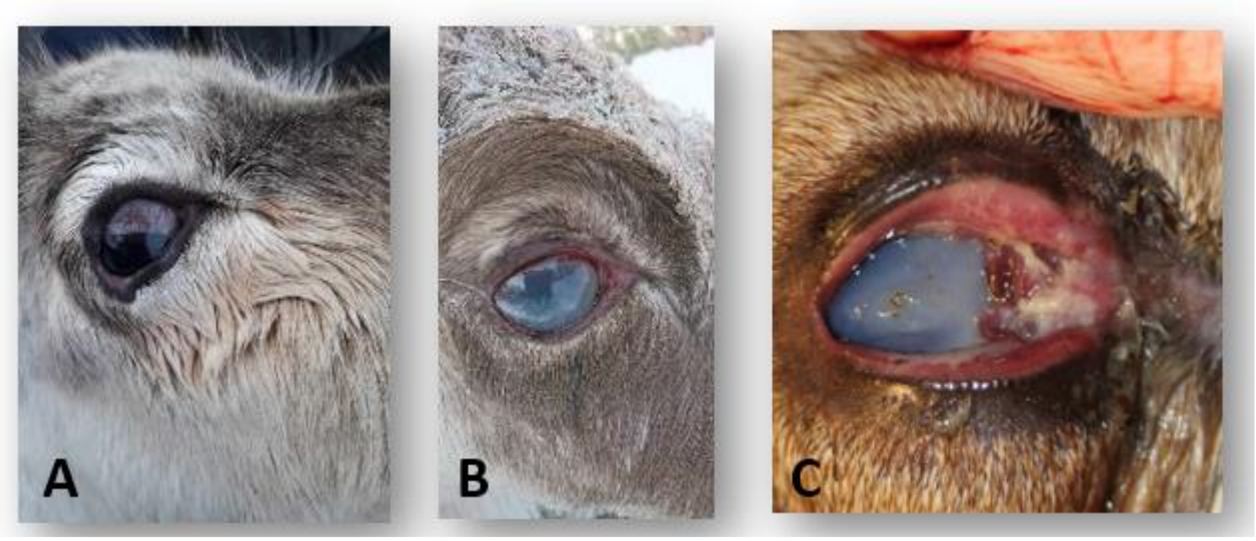

A: Tårflöde som ger blöt kind, tidigt stadie.

B: Blå-grå hornhinna och röd slemhinna, tilltagande symtom.

C: Röd slemhinna, svullet öga och varigt tårflöde, tilltagande symtom.

9) * Har du sett liknande ögonförändringar som Bild 1. A-C visar på dina renar under de senaste 10 åren?

- ○ Ja
- ○ Nej
- ○ Vet ej / annan ögonförändring, beskriv nedan

10) Beskriv vilken annan ögonförändring du sett:

Denna informationsbox visas endast i läge förhandsgranskningen.

Följande kriterium måste vara uppfyllda för att följande fråga ska visas:

Om frågan Har du sett liknande ögonförändringar som Bild 1. A-C visar på dina renar under de senaste 10 åren? innehåller något av dessa svarsalternativ

- Vet ej / annan ögonförändring, beskriv nedan
- Ja

Smittsam ögoninflammation/ögonförändring

Bild 1. A-C:

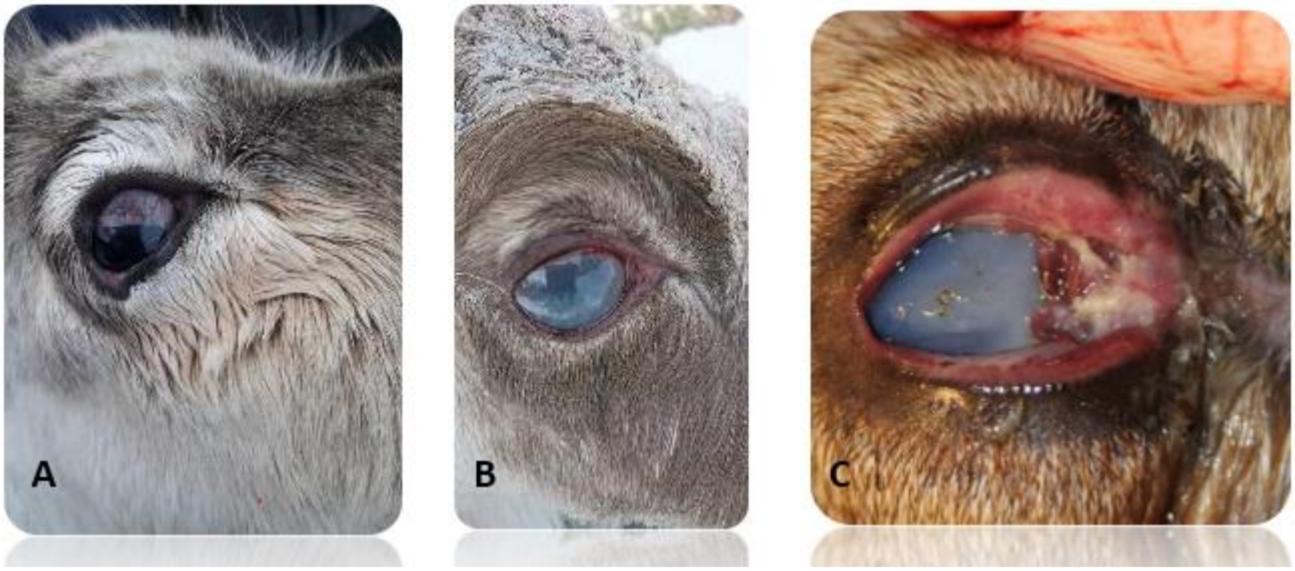

11) När såg du senast liknande ögonförändringar som Bild 1. A-C visar på dina renar? Om du angivit en annan ögonförändring/symtom svarar du utifrån den på följande frågor om ögoninflammation/ögonförändring.

- ◯ Senaste året
- ◯ Ej under senaste året men under de senaste 5 åren
- ◯ Mer än 5 år sedan
- ◯ Vet ej

Denna informationsbox visas endast i läge förhandsgranskningen.

Följande kriterium måste vara uppfyllda för att följande fråga ska visas:

Om frågan Har du sett liknande ögonförändringar som Bild 1. A-C visar på dina renar under de senaste 10 åren? innehåller något av dessa svarsalternativ

- Vet ej / annan ögonförändring, beskriv nedan
- Ja

Smittsam ögoninflammation/ögonförändring

Bild 1. A-C:

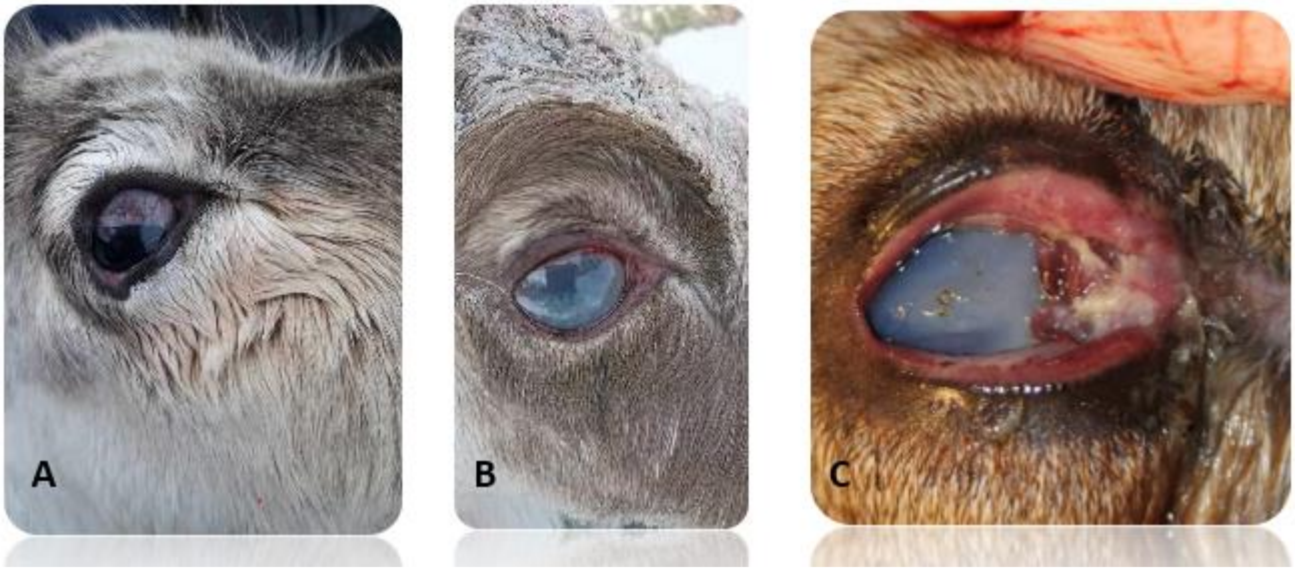

12) Vilken av ögonförändringarna, som bilderna 1. A-C visar, ser du vanligtvis på dina drabbade renar?

	Aldrig	Ibland	Ofta
A			
B			
C			
Annat symtom, beskriv i kommentarer nedan			

Denna informationsbox visas endast i läge förhandsgranskningen.

Följande kriterium måste vara uppfyllda för att följande fråga ska visas:

Om frågan Har du sett liknande ögonförändringar som Bild 1. A-C visar på dina renar under de senaste 10 åren? innehåller något av dessa svarsalternativ

- Vet ej / annan ögonförändring, beskriv nedan

- Ja

13) Plats för kommentarer:

Denna informationsbox visas endast i läge förhandsgranskningen.

Följande kriterium måste vara uppfyllda för att följande fråga ska visas:

Om frågan Har du sett liknande ögonförändringar som Bild 1. A-C visar på dina renar under de senaste 10 åren? innehåller något av dessa svarsalternativ

- Vet ej / annan ögonförändring, beskriv nedan
- Ja

Smittsam ögoninflammation/ögonförändring

14) När på året ser du flest antal renar med smittsam ögoninflammation/ögonförändring? Ange för kalv, unga och vuxna djur. Det är möjligt att välja flera årstider.

	Ej observerat	Vår	Sommar	Höst	Vinter	Året runt (anges som enda alternativ!)
Kalv (yngre än 1 år)	☐	☐	☐	☐	☐	☐
Unga djur (1-3 år)	☐	☐	☐	☐	☐	☐
Vuxna djur (äldre än 3 år)	☐	☐	☐	☐	☐	☐

Denna informationsbox visas endast i läge förhandsgranskningen.

Följande kriterium måste vara uppfyllda för att följande fråga ska visas:

Om frågan Har du sett liknande ögonförändringar som Bild 1. A-C visar på dina renar under de senaste 10 åren? innehåller något av dessa svarsalternativ

- Vet ej / annan ögonförändring, beskriv nedan
- Ja

15) Plats för kommentarer:

Denna informationsbox visas endast i läge förhandsgranskningen.

Följande kriterium måste vara uppfyllda för att följande fråga ska visas:

Om frågan Har du sett liknande ögonförändringar som Bild 1. A-C visar på dina renar under de senaste 10 åren? innehåller något av dessa svarsalternativ

- Vet ej / annan ögonförändring, beskriv nedan
- Ja

Smittsam ögoninflammation/ögonförändring

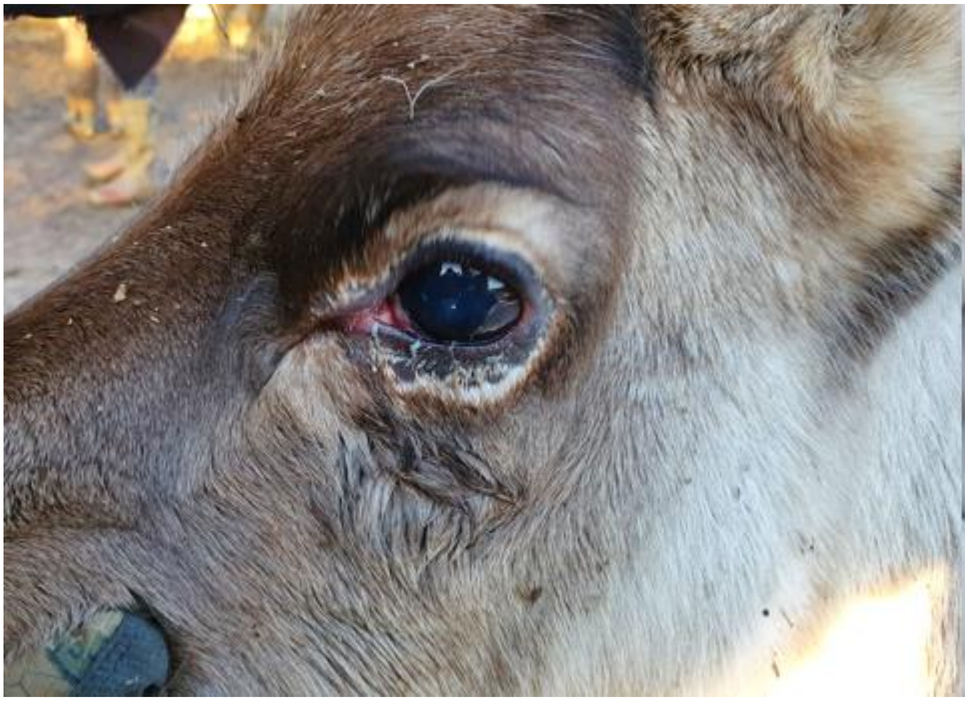

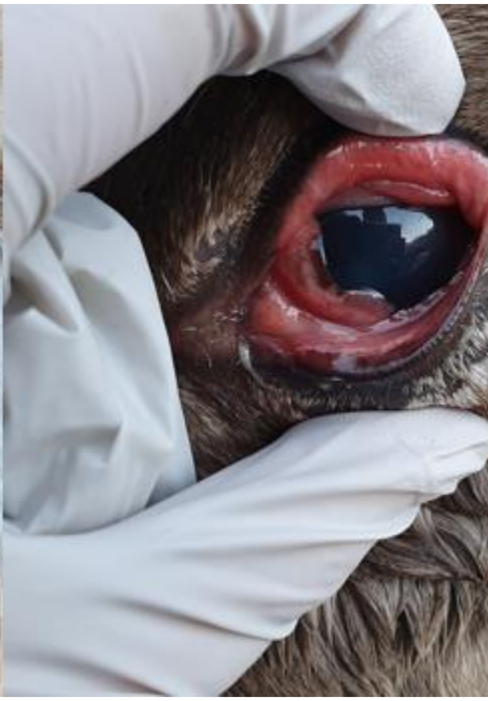

Ren med tårflöde. *Renen är svullen och röd i ögat vid närmre undersökning.*

16) Ange, för var och en av nedanstående säsonger, var renarna befann sig under utbrott av smittsam ögoninflammation/ögonförändring alternativt att inget utbrott ägde rum. Utbrott definieras som en tydlig ökning av antalet fall under en viss tidsperiod, till

exempel fler drabbade djur än vanligt under en säsong, eller under en kortare tidsperiod, exempelvis några veckor.

	Ej upplevt utbrott	Utbrott i hägn	Utbrott på fribete med utfodring	Utbrott på fribete utan utfodring
2015/2016	☐	☐	☐	☐
2016/2017	☐	☐	☐	☐
2017/2018	☐	☐	☐	☐
2018/2019	☐	☐	☐	☐
2019/2020	☐	☐	☐	☐
2020/2021	☐	☐	☐	☐

Denna informationsbox visas endast i läge förhandsgranskningen.

Följande kriterium måste vara uppfyllda för att följande fråga ska visas:

Om frågan Har du sett liknande ögonförändringar som Bild 1. A-C visar på dina renar under de senaste 10 åren? innehåller något av dessa svarsalternativ

- Vet ej / annan ögonförändring, beskriv nedan
- Ja

17) Plats för kommentarer:

Denna informationsbox visas endast i läge förhandsgranskningen.

Följande kriterium måste vara uppfyllda för att följande fråga ska visas:

Om frågan Har du sett liknande ögonförändringar som Bild 1. A-C visar på dina renar under de senaste 10 åren? innehåller något av dessa svarsalternativ

- Vet ej / annan ögonförändring, beskriv nedan
- Ja

Smittsam ögoninflammation/ögonförändring

18) Ungefär hur många renar drabbades i gruppen där det senast var utbrott, alternativt fall av, smittsam ögoninflammation/ögonförändring? Ange i första hand antalet vid utbrott om du upplevt det.

Kalv (yngre än 1 år)

Unga djur (1-3 år)

Vuxna djur (äldre än 3 år)

Totalt antal renar i gruppen (både drabbade och friska)

Denna informationsbox visas endast i läge förhandsgranskningen.

Följande kriterium måste vara uppfyllda för att följande fråga ska visas:

Om frågan Har du sett liknande ögonförändringar som Bild 1. A-C visar på dina renar under de senaste 10 åren? innehåller något av dessa svarsalternativ

- Vet ej / annan ögonförändring, beskriv nedan
- Ja

19) Plats för kommentarer:

Denna informationsbox visas endast i läge förhandsgranskningen.

Följande kriterium måste vara uppfyllda för att följande fråga ska visas:

Om frågan Har du sett liknande ögonförändringar som Bild 1. A-C visar på dina renar under de senaste 10 åren? innehåller något av dessa svarsalternativ

- Vet ej / annan ögonförändring, beskriv nedan
- Ja

Smittsam ögoninflammation/ögonförändring

20) * Upplever du att antalet renar som drabbats av smittsam ögoninflammation/ögonförändring har förändrats över de senaste fem åren?

- Ja, förekomsten har ökat
- Ja, förekomsten har minskat
- Nej, förekomsten har varken ökat eller minskat
- Vet ej

Denna informationsbox visas endast i läge förhandsgranskningen.

Följande kriterium måste vara uppfyllda för att följande fråga ska visas:

Om frågan Har du sett liknande ögonförändringar som Bild 1. A-C visar på dina renar under de senaste 10 åren? innehåller något av dessa svarsalternativ

- Vet ej / annan ögonförändring, beskriv nedan
- Ja

och

Om frågan Upplever du att antalet renar som drabbats av smittsam ögoninflammation/ögonförändring har förändrats över de senaste fem åren? innehåller något av dessa svarsalternativ

- Ja, förekomsten har ökat

Smittsam ögoninflammation/ögonförändring

21) Vad tror du att denna ökning av antalet drabbade renar kan beror på?

Denna informationsbox visas endast i läge förhandsgranskningen.

Följande kriterium måste vara uppfyllda för att följande fråga ska visas:

Om frågan Har du sett liknande ögonförändringar som Bild 1. A-C visar på dina renar under de senaste 10 åren? innehåller något av dessa svarsalternativ

- Vet ej / annan ögonförändring, beskriv nedan
- Ja

och

Om frågan Upplever du att antalet renar som drabbats av smittsam ögoninflammation/ögonförändring har förändrats över de senaste fem åren? innehåller något av dessa svarsalternativ

- Ja, förekomsten har minskat

Smittsam ögoninflammation/ögonförändring

22) Vad tror du att denna minskning av antalet drabbade renar kan beror på?

Denna informationsbox visas endast i läge förhandsgranskningen.

Följande kriterium måste vara uppfyllda för att följande fråga ska visas:

Om frågan Har du sett liknande ögonförändringar som Bild 1. A-C visar på dina renar under de senaste 10 åren? innehåller något av dessa svarsalternativ

- Vet ej / annan ögonförändring, beskriv nedan
- Ja

Smittsam ögoninflammation/ögonförändring

23) * Vidtas vanligtvis åtgärder (exempelvis gruppering, slakt eller annan behandling) när du ser renar drabbade av smittsam ögoninflammation/ögonförändring?

- ○ Ja
- ○ Nej
- ○ Vet ej

Denna informationsbox visas endast i läge förhandsgranskningen.

Följande kriterium måste vara uppfyllda för att följande fråga ska visas:

Om frågan Har du sett liknande ögonförändringar som Bild 1. A-C visar på dina renar under de senaste 10 åren? innehåller något av dessa svarsalternativ

- Vet ej / annan ögonförändring, beskriv nedan

- Ja

24) Vilka åtgärder sattes in när du senast såg renar drabbade av smittsam ögoninflammation/ögonförändring? Ange alla åtgärder som sattes in.

☐Ingen åtgärd
☐Gruppering av drabbade djur i sjukhägn
☐Veterinär kontaktades
☐Antibiotika som ges med spruta i muskeln
☐Antibiotika i ögat
☐Slakt
☐Avlivning/kassering
☐Behandling med övrigt läkemedel, ange vad i kommentarer nedan
☐Annat, ange vad i kommentarer nedan

Denna informationsbox visas endast i läge förhandsgranskningen.

Följande kriterium måste vara uppfyllda för att följande fråga ska visas:

Om frågan Har du sett liknande ögonförändringar som Bild 1. A-C visar på dina renar under de senaste 10 åren? innehåller något av dessa svarsalternativ

- Vet ej / annan ögonförändring, beskriv nedan
- Ja

25) Plats för kommentarer:

Denna informationsbox visas endast i läge förhandsgranskningen.

Följande kriterium måste vara uppfyllda för att följande fråga ska visas:

Om frågan Har du sett liknande ögonförändringar som Bild 1. A-C visar på dina renar under de senaste 10 åren? innehåller något av dessa svarsalternativ

- Vet ej / annan ögonförändring, beskriv nedan
- Ja

Smittsam ögoninflammation/ögonförändring

26) Har utbrott eller fall av smittsam ögoninflammation/ögonförändring medfört ekonomiska konsekvenser?

- Ja
- Nej
- Vet ej

Denna informationsbox visas endast i läge förhandsgranskningen.

Följande kriterium måste vara uppfyllda för att följande fråga ska visas:

Om frågan Har du sett liknande ögonförändringar som Bild 1. A-C visar på dina renar under de senaste 10 åren? innehåller något av dessa svarsalternativ

- Vet ej / annan ögonförändring, beskriv nedan
- Ja

27) Plats för kommentarer:

Denna informationsbox visas endast i läge förhandsgranskningen.

Följande kriterium måste vara uppfyllda för att följande fråga ska visas:

Om frågan Har du sett liknande ögonförändringar som Bild 1. A-C visar på dina renar under de senaste 10 åren? innehåller något av dessa svarsalternativ

- Vet ej / annan ögonförändring, beskriv nedan
- Ja

Smittsam ögoninflammation/ögonförändring

Bild 1. A-C:

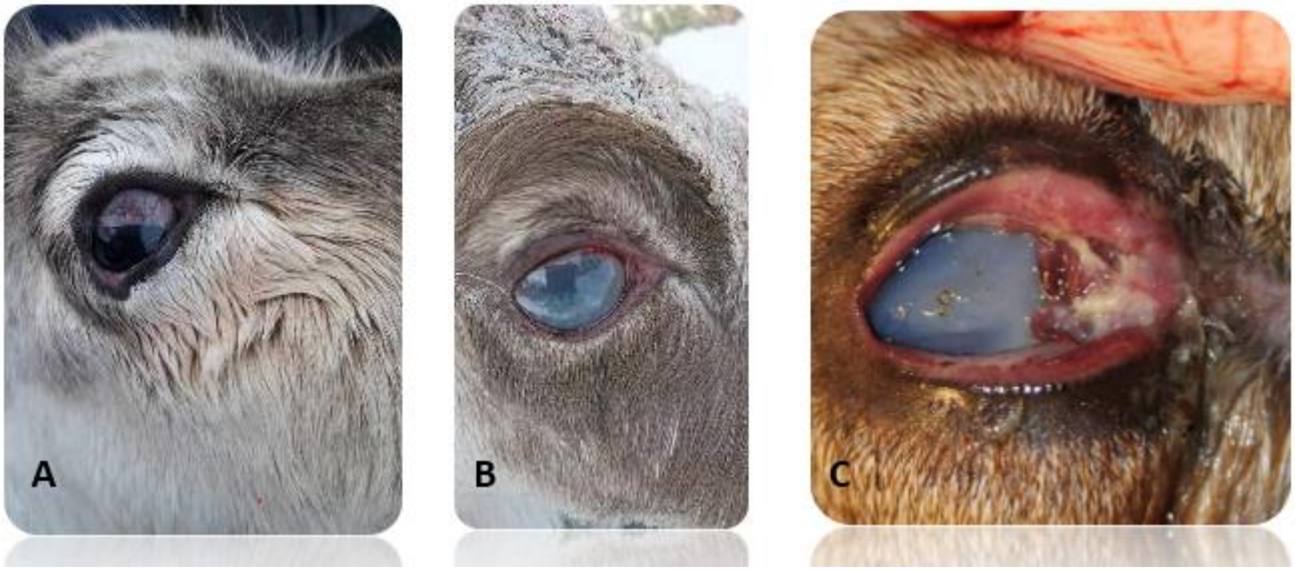

28) Har någon av förändringarna på bilderna ett för dig eller för samebyn traditionellt namn?

- ○ Ja, ange i kommentarsfältet nedan
- ○ Nej
- ○ Vet ej

Denna informationsbox visas endast i läge förhandsgranskningen.

Följande kriterium måste vara uppfyllda för att följande fråga ska visas:

Om frågan Har du sett liknande ögonförändringar som Bild 1. A-C visar på dina renar under de senaste 10 åren? innehåller något av dessa svarsalternativ

- Vet ej / annan ögonförändring, beskriv nedan
- Ja

29) Plats för kommentarer/ange traditionellt namn:

A:

B:

C:

Annan ögonförändring:

Denna informationsbox visas endast i läge förhandsgranskningen.

Följande kriterium måste vara uppfyllda för att följande fråga ska visas:

Om frågan Har du sett liknande ögonförändringar som Bild 1. A-C visar på dina renar under de senaste 10 åren? innehåller något av dessa svarsalternativ

- Vet ej / annan ögonförändring, beskriv nedan
- Ja

30) Känner du till någon traditionell behandling av smittsam ögoninflammation/ögonförändring?

- Ja, beskriv i kommentarsfältet nedan
- Nej
- Vet ej

Denna informationsbox visas endast i läge förhandsgranskningen.

Följande kriterium måste vara uppfyllda för att följande fråga ska visas:

Om frågan Har du sett liknande ögonförändringar som Bild 1. A-C visar på dina renar under de senaste 10 åren? innehåller något av dessa svarsalternativ

- Vet ej / annan ögonförändring, beskriv nedan
- Ja

31) Plats för kommentarer:

Denna informationsbox visas endast i läge förhandsgranskningen.

Följande kriterium måste vara uppfyllda för att följande fråga ska visas:

Om frågan Har du sett liknande ögonförändringar som Bild 1. A-C visar på dina renar under de senaste 10 åren? innehåller något av dessa svarsalternativ

- Vet ej / annan ögonförändring, beskriv nedan
- Nej
- Ja

Strax följer frågor om munvårtsjuka/orf

32) Lämna gärna övriga kommentarer kopplade till smittsam ögoninflammation/ögonförändring här:

2. Här följer några frågor om munvårtsjuka/orf:

Bild 2. A-B:

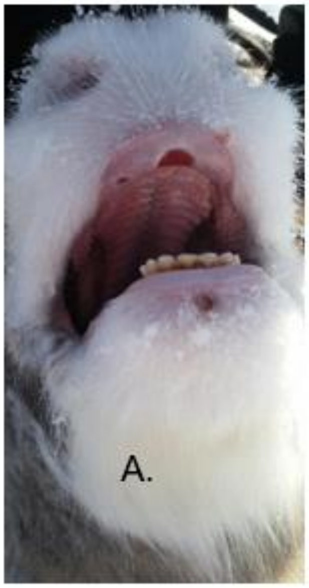

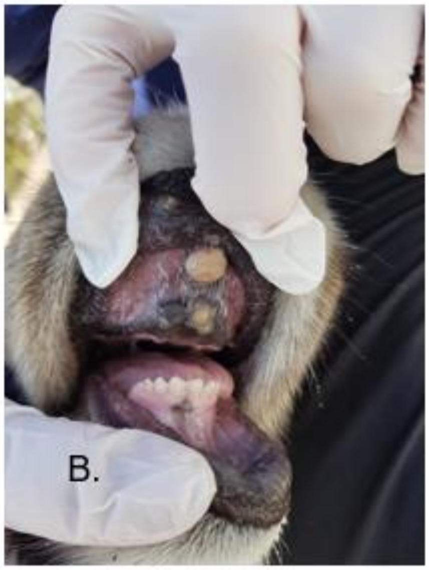

A: Sprucken blåsa i munhåla och läpp orsakad av orfvirus, tidigt stadie.

B: Blåsor i munhålan och tandkött, senare stadie.

33) * Har du sett liknande förändringar som Bild 2. A-B visar på dina renar under de senaste 10 åren?

- Ja
- Nej
- Vet ej

Denna informationsbox visas endast i läge förhandsgranskningen.

Följande kriterium måste vara uppfyllda för att följande fråga ska visas:

Om frågan Har du sett liknande förändringar som Bild 2. A-B visar på dina renar under de senaste 10 åren? innehåller något av dessa svarsalternativ

- Vet ej
- Ja

Munvårtsjuka/orf

Bild 2. A-B:

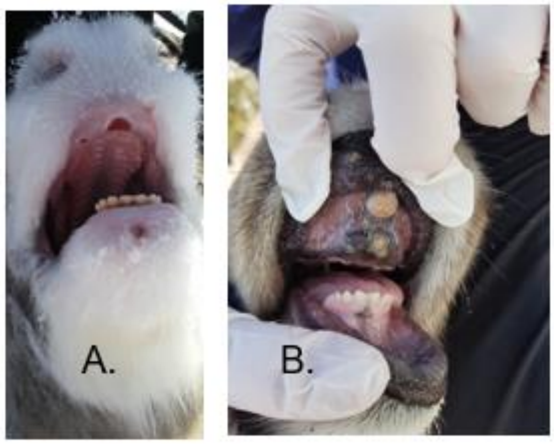

34) När såg du senast liknande förändringar som Bild 2. A-B visar på dina renar?

- ○ Senaste året
- ○ Ej under senaste året men under de senaste 5 åren
- ○ Mer än 5 år sedan
- ○ Vet ej

Denna informationsbox visas endast i läge förhandsgranskningen.

Följande kriterium måste vara uppfyllda för att följande fråga ska visas:

Om frågan Har du sett liknande förändringar som Bild 2. A-B visar på dina renar under de senaste 10 åren? innehåller något av dessa svarsalternativ

- Vet ej
- Ja

Munvårtsjuka/orf

35) När på året ser du flest antal renar med munvårtsjuka/orf? Ange för kalv, unga och vuxna djur. Det är möjligt att välja flera årstider.

	Ej observerat	Vår	Sommar	Höst	Vinter	Året runt (anges som enda svarsalternativ!)
Kalv (yngre än 1 år)	☐	☐	☐	☐	☐	☐
Unga djur (1-3 år)	☐	☐	☐	☐	☐	☐
Vuxna djur (äldre än 3 år)	☐	☐	☐	☐	☐	☐

Denna informationsbox visas endast i läge förhandsgranskningen.

Följande kriterium måste vara uppfyllda för att följande fråga ska visas:

Om frågan Har du sett liknande förändringar som Bild 2. A-B visar på dina renar under de senaste 10 åren? innehåller något av dessa svarsalternativ

- Vet ej
- Ja

36) Plats för kommentarer:

Denna informationsbox visas endast i läge förhandsgranskningen.

Följande kriterium måste vara uppfyllda för att följande fråga ska visas:

Om frågan Har du sett liknande förändringar som Bild 2. A-B visar på dina renar under de senaste 10 åren? innehåller något av dessa svarsalternativ

- Vet ej
- Ja

Munvårtsjuka/orf

37) Ange, för var och en av nedanstående säsonger, var renarna befann sig under utbrott av munvårtsjuk/orf alternativt att inget utbrott ägde rum. Utbrott av sjukdom definieras som en tydlig ökning av antalet fall under en viss tidsperiod, till exempel fler drabbade djur än vanligt under en säsong, eller under en kortare tidsperiod, exempelvis några veckor.

	Ej upplevt utbrott	Utbrott i hägn	Utbrott på fribete med utfodring	Utbrott på fribete utan utfodring
2015/2016	☐	☐	☐	☐
2016/2017	☐	☐	☐	☐
2017/2018	☐	☐	☐	☐
2018/2019	☐	☐	☐	☐
2019/2020	☐	☐	☐	☐
2020/2021	☐	☐	☐	☐

Denna informationsbox visas endast i läge förhandsgranskningen.

Följande kriterium måste vara uppfyllda för att följande fråga ska visas:

Om frågan Har du sett liknande förändringar som Bild 2. A-B visar på dina renar under de senaste 10 åren? innehåller något av dessa svarsalternativ

- Vet ej
- Ja

38) Plats för kommentarer:

Denna informationsbox visas endast i läge förhandsgranskningen.

Följande kriterium måste vara uppfyllda för att följande fråga ska visas:

Om frågan Har du sett liknande förändringar som Bild 2. A-B visar på dina renar under de senaste 10 åren? innehåller något av dessa svarsalternativ

- Vet ej
- Ja

Munvårtsjuka/orf

39) Ungefär hur många renar drabbades i gruppen där det senast var utbrott, alternativt fall av, munvårtsjuka/orf? Ange i första hand antalet vid utbrott om du upplevt det.

Kalv (yngre än 1 år)

Unga djur (1-3 år)

Vuxna djur (äldre än 3 år)

Totalt antal renar i gruppen (både drabbade och friska)

Denna informationsbox visas endast i läge förhandsgranskningen.

Följande kriterium måste vara uppfyllda för att följande fråga ska visas:

Om frågan Har du sett liknande förändringar som Bild 2. A-B visar på dina renar under de senaste 10 åren? innehåller något av dessa svarsalternativ

- Vet ej
- Ja

40) Plats för kommentarer:

Denna informationsbox visas endast i läge förhandsgranskningen.

Följande kriterium måste vara uppfyllda för att följande fråga ska visas:

Om frågan Har du sett liknande förändringar som Bild 2. A-B visar på dina renar under de senaste 10 åren? innehåller något av dessa svarsalternativ

- Vet ej
- Ja

Munvårtsjuka/orf

41) * Upplever du att antalet renar som drabbats av munvårtsjuka/orf har förändrats över de senaste 5 åren?

- ○ Ja, förekomsten har ökat
- ○ Ja, förekomsten har minskat
- ○ Nej, förekomsten har varken ökat eller minskat.
- ○ Vet ej

Denna informationsbox visas endast i läge förhandsgranskningen.

Följande kriterium måste vara uppfyllda för att följande fråga ska visas:

Om frågan Har du sett liknande förändringar som Bild 2. A-B visar på dina renar under de senaste 10 åren? innehåller något av dessa svarsalternativ

- Vet ej
- Ja

och

Om frågan Upplever du att antalet renar som drabbats av munvårtsjuka/orf har förändrats över de senaste 5 åren? innehåller något av dessa svarsalternativ

- Ja, förekomsten har ökat

Munvårtsjuka/orf

42) Vad tror du att denna ökning av antalet drabbade renar kan beror på?

Denna informationsbox visas endast i läge förhandsgranskningen.

Följande kriterium måste vara uppfyllda för att följande fråga ska visas:

Om frågan Har du sett liknande förändringar som Bild 2. A-B visar på dina renar under de senaste 10 åren? innehåller något av dessa svarsalternativ

- Vet ej

- Ja

och

Om frågan Upplever du att antalet renar som drabbats av munvårtsjuka/orf har förändrats över de senaste 5 åren? innehåller något av dessa svarsalternativ

- Ja, förekomsten har minskat

Munvårtsjuka/orf

43) Vad tror du att denna minskning av antalet drabbade renar kan beror på?

Denna informationsbox visas endast i läge förhandsgranskningen.

Följande kriterium måste vara uppfyllda för att följande fråga ska visas:

Om frågan Har du sett liknande förändringar som Bild 2. A-B visar på dina renar under de senaste 10 åren? innehåller något av dessa svarsalternativ

- Vet ej
- Ja

Munvårtsjuka/orf

44) * Vidtas vanligtvis åtgärder (exempelvis gruppering, slakt eller annan behandling) när du ser renar drabbade av munvårtsjuka/orf?

- ○ Ja
- ○ Nej
- ○ Vet ej

Denna informationsbox visas endast i läge förhandsgranskningen.

Följande kriterium måste vara uppfyllda för att följande fråga ska visas:

Om frågan Har du sett liknande förändringar som Bild 2. A-B visar på dina renar under de senaste 10 åren? innehåller något av dessa svarsalternativ

- Vet ej

- Ja

45) Vilka åtgärder sattes in när du senast såg renar drabbade av munvårtsjuka/orf? Ange alla åtgärder som sattes in.

- [ ] Ingen åtgärd
- [ ] Gruppering av drabbade djur i sjukhägn
- [ ] Veterinär kontaktades
- [ ] Antibiotikabehandling
- [ ] Slakt
- [ ] Avlivning/kassering
- [ ] Behandling med övrigt läkemedel, ange vad i kommentarer nedan
- [ ] Annat, ange vad i kommentarer nedan

Denna informationsbox visas endast i läge förhandsgranskningen.

Följande kriterium måste vara uppfyllda för att följande fråga ska visas:

Om frågan Har du sett liknande förändringar som Bild 2. A-B visar på dina renar under de senaste 10 åren? innehåller något av dessa svarsalternativ

- Vet ej
- Ja

46) Plats för kommentarer:

Denna informationsbox visas endast i läge förhandsgranskningen.

Följande kriterium måste vara uppfyllda för att följande fråga ska visas:

Om frågan Har du sett liknande förändringar som Bild 2. A-B visar på dina renar under de senaste 10 åren? innehåller något av dessa svarsalternativ

- Vet ej
- Ja

Munvårtsjuka/orf

47) Har utbrott eller fall av munvårtsjuka/orf medfört ekonomiska konsekvenser?

- ○ Ja
- ○ Nej
- ○ Vet ej

Denna informationsbox visas endast i läge förhandsgranskningen.

Följande kriterium måste vara uppfyllda för att följande fråga ska visas:

Om frågan Har du sett liknande förändringar som Bild 2. A-B visar på dina renar under de senaste 10 åren? innehåller något av dessa svarsalternativ

- Vet ej
- Ja

48) Plats för kommentarer:

Denna informationsbox visas endast i läge förhandsgranskningen.

Följande kriterium måste vara uppfyllda för att följande fråga ska visas:

Om frågan Har du sett liknande förändringar som Bild 2. A-B visar på dina renar under de senaste 10 åren? innehåller något av dessa svarsalternativ

- Vet ej
- Ja

Munvårtsjuka/orf

Bild 2. A-B:

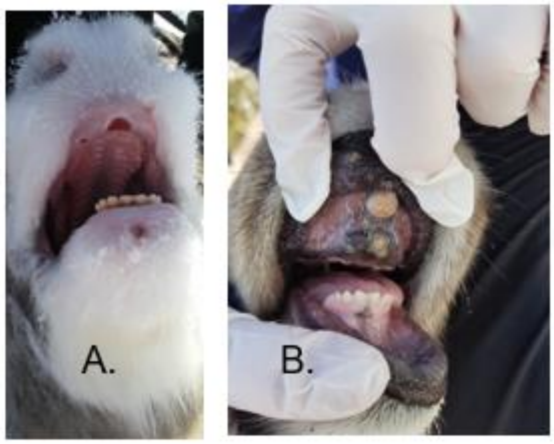

49) Har förändringarna på bilderna ett för dig eller för samebyn traditionellt namn?

- Ja, ange i kommentarsfältet nedan
- Nej
- Vet ej

Denna informationsbox visas endast i läge förhandsgranskningen.

Följande kriterium måste vara uppfyllda för att följande fråga ska visas:

Om frågan Har du sett liknande förändringar som Bild 2. A-B visar på dina renar under de senaste 10 åren? innehåller något av dessa svarsalternativ

- Vet ej
- Ja

50) Plats för kommentarer/ange traditionellt namn:

Denna informationsbox visas endast i läge förhandsgranskningen.

Följande kriterium måste vara uppfyllda för att följande fråga ska visas:

Om frågan Har du sett liknande förändringar som Bild 2. A-B visar på dina renar under de senaste 10 åren? innehåller något av dessa svarsalternativ

- Vet ej
- Ja

51) Känner du till någon traditionell behandling av munvårtsjuka/orf ?

- ○ Ja, beskriv i kommentarsfältet nedan
- ○ Nej
- ○ Vet ej

Denna informationsbox visas endast i läge förhandsgranskningen.

Följande kriterium måste vara uppfyllda för att följande fråga ska visas:

Om frågan Har du sett liknande förändringar som Bild 2. A-B visar på dina renar under de senaste 10 åren? innehåller något av dessa svarsalternativ

- Vet ej
- Ja

52) Plats för kommentarer:

Denna informationsbox visas endast i läge förhandsgranskningen.

Följande kriterium måste vara uppfyllda för att följande fråga ska visas:

Om frågan Har du sett liknande förändringar som Bild 2. A-B visar på dina renar under de senaste 10 åren? innehåller något av dessa svarsalternativ

- Vet ej
- Nej
- Ja

Strax följer frågor om munröta/oral nekrobacillos/njunnevikke

53) Lämna gärna övriga kommentarer kopplade till munvårtsjuka/orf här:

3. Frågor om munröta/oral nekrobacillos/njunnevikke :

Bild 3. A-C:

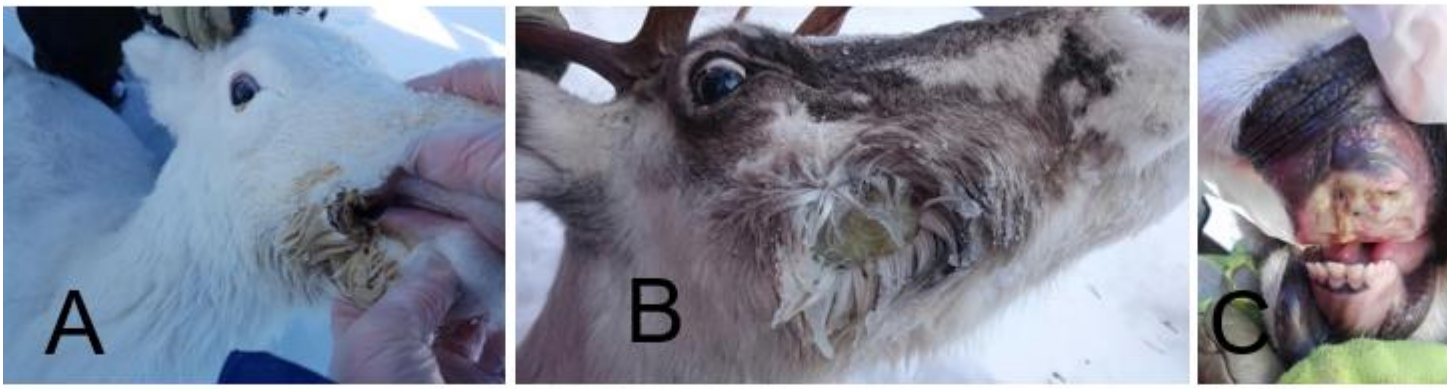

A: Sårig och kladdig mungipa med varbildning.

B: Infektion i kinden där ett varigt 'hål' i huden har bildats.

C: Infektion i tandköttet med varbildning.

54) * Har du sett liknande förändringar som Bild 3. A-C visar på dina renar under de senaste 10 åren?

- ○ Ja
- ○ Nej
- ○ Vet ej

Denna informationsbox visas endast i läge förhandsgranskningen.

Följande kriterium måste vara uppfyllda för att följande fråga ska visas:

Om frågan Har du sett liknande förändringar som Bild 3. A-C visar på dina renar under de senaste 10 åren? innehåller något av dessa svarsalternativ

- Vet ej
- Ja

Munröta/oral nekrobacillos/njunnevikke

Bild 3. A-C:

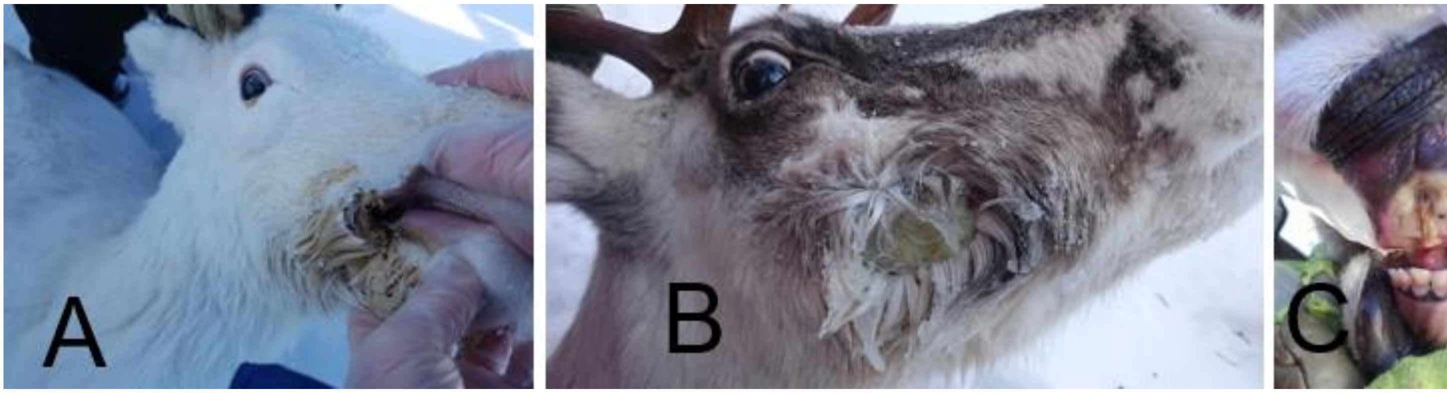

55) När såg du senast liknande förändringar som Bild 3. A-C visar på dina renar?

- ○ Senaste året
- ○ Ej under senaste året men under de senaste 5 åren
- ○ Mer än 5 år sedan
- ○ Vet ej

Denna informationsbox visas endast i läge förhandsgranskningen.

Följande kriterium måste vara uppfyllda för att följande fråga ska visas:

Om frågan Har du sett liknande förändringar som Bild 3. A-C visar på dina renar under de senaste 10 åren? innehåller något av dessa svarsalternativ

- Vet ej
- Ja

Munröta/oral nekrobacillos/njunnevikke

56) När på året ser du flest antal renar med symtom på munröta/oral nekrobacillos/njunnevikke? Ange för kalv, unga och vuxna djur. Det är möjligt att välja flera årstider.

	Ej observerat	Vår	Sommar	Höst	Vinter	Året runt (anges som enda svarsalternativ!)
Kalv (yngre än 1 år)	☐	☐	☐	☐	☐	☐
Unga djur (1-3 år)	☐	☐	☐	☐	☐	☐
Vuxna djur (äldre än 3 år)	☐	☐	☐	☐	☐	☐

Denna informationsbox visas endast i läge förhandsgranskningen.

Följande kriterium måste vara uppfyllda för att följande fråga ska visas:

Om frågan Har du sett liknande förändringar som Bild 3. A-C visar på dina renar under de senaste 10 åren? innehåller något av dessa svarsalternativ

- Vet ej
- Ja

57) Plats för kommentarer:

Denna informationsbox visas endast i läge förhandsgranskningen.

Följande kriterium måste vara uppfyllda för att följande fråga ska visas:

Om frågan Har du sett liknande förändringar som Bild 3. A-C visar på dina renar under de senaste 10 åren? innehåller något av dessa svarsalternativ

- Vet ej
- Ja

Munröta/oral nekrobacillos/njunnevikke

58) Ange, för var och en av nedanstående säsonger, var renarna befann sig under utbrott av munröta/oral nekrobacillos/njunnevikke alternativt att inget utbrott ägde rum. Utbrott av sjukdom definieras som en tydlig ökning av antalet fall under en viss tidsperiod, till exempel fler drabbade djur än vanligt under en säsong, eller under en kortare tidsperiod, exempelvis några veckor.

	Ej upplevt utbrott	Utbrott i hägn	Utbrott på fribete med utfodring	Utbrott på fribete utan utfodring
2015/2016	☐	☐	☐	☐
2016/2017	☐	☐	☐	☐
2017/2018	☐	☐	☐	☐
2018/2019	☐	☐	☐	☐
2019/2020	☐	☐	☐	☐
2020/2021	☐	☐	☐	☐

Denna informationsbox visas endast i läge förhandsgranskningen.

Följande kriterium måste vara uppfyllda för att följande fråga ska visas:

Om frågan Har du sett liknande förändringar som Bild 3. A-C visar på dina renar under de senaste 10 åren? innehåller något av dessa svarsalternativ

- Vet ej
- Ja

59) Plats för kommentarer:

Denna informationsbox visas endast i läge förhandsgranskningen.

Följande kriterium måste vara uppfyllda för att följande fråga ska visas:

Om frågan Har du sett liknande förändringar som Bild 3. A-C visar på dina renar under de senaste 10 åren? innehåller något av dessa svarsalternativ

- Vet ej
- Ja

Munröta/oral nekrobacillos/njunnevikke

60) Ungefär hur många renar drabbades i gruppen där det senaste var utbrott eller fall av munröta/oral nekrobacillos/njunnevikke? Ange i första hand antalet vid utbrott om du upplevt det.

Kalv (yngre än 1 år)

Unga djur (1-3 år)

Vuxna djur (äldre än 3 år)

Totalt antal renar i gruppen (både drabbade och friska)

Denna informationsbox visas endast i läge förhandsgranskningen.

Följande kriterium måste vara uppfyllda för att följande fråga ska visas:

Om frågan Har du sett liknande förändringar som Bild 3. A-C visar på dina renar under de senaste 10 åren? innehåller något av dessa svarsalternativ

- Vet ej
- Ja

61) Plats för kommentarer:

Denna informationsbox visas endast i läge förhandsgranskningen.

Följande kriterium måste vara uppfyllda för att följande fråga ska visas:

Om frågan Har du sett liknande förändringar som Bild 3. A-C visar på dina renar under de senaste 10 åren? innehåller något av dessa svarsalternativ

- Vet ej
- Ja

Munröta/oral nekrobacillos/njunnevikke

62) * Upplever du att antalet renar som drabbats av munröta/oral nekrobacillos/njunnevikke har förändrats över de senaste fem åren?

- ○ Ja, förekomsten har ökat

- ◯ Ja, förekomsten har minskat
- ◯ Nej, förekomsten har varken ökat eller minskat
- ◯ Vet ej

Denna informationsbox visas endast i läge förhandsgranskningen.

Följande kriterium måste vara uppfyllda för att följande fråga ska visas:

Om frågan Har du sett liknande förändringar som Bild 3. A-C visar på dina renar under de senaste 10 åren? innehåller något av dessa svarsalternativ

- Vet ej
- Ja

och

Om frågan Upplever du att antalet renar som drabbats av munröta/oral nekrobacillos/njunnevikke har förändrats över de senaste fem åren? innehåller något av dessa svarsalternativ

- Ja, förekomsten har ökat

Munröta/oral nekrobacillos/njunnevikke

63) Vad tror du att denna ökning av antalet drabbade renar kan beror på?

Denna informationsbox visas endast i läge förhandsgranskningen.

Följande kriterium måste vara uppfyllda för att följande fråga ska visas:

Om frågan Har du sett liknande förändringar som Bild 3. A-C visar på dina renar under de senaste 10 åren? innehåller något av dessa svarsalternativ

- Vet ej
- Ja

och

Om frågan Upplever du att antalet renar som drabbats av munröta/oral nekrobacillos/njunnevikke har förändrats över de senaste fem åren? innehåller något av dessa svarsalternativ

- Ja, förekomsten har minskat

Munröta/oral nekrobacillos/njunnevikke

64) Vad tror du att denna minskning av antalet drabbade renar kan beror på?

Denna informationsbox visas endast i läge förhandsgranskningen.

Följande kriterium måste vara uppfyllda för att följande fråga ska visas:

Om frågan Har du sett liknande förändringar som Bild 3. A-C visar på dina renar under de senaste 10 åren? innehåller något av dessa svarsalternativ

- Vet ej
- Ja

Munröta/oral nekrobacillos/njunnevikke

65) * Vidtas vanligtvis åtgärder (exempelvis gruppering, slakt eller annan behandling) när du ser renar drabbade av munröta/oral nekrobacillos/njunnevikke?

○ Ja
○ Nej
○ Vet ej

Denna informationsbox visas endast i läge förhandsgranskningen.

Följande kriterium måste vara uppfyllda för att följande fråga ska visas:

Om frågan Har du sett liknande förändringar som Bild 3. A-C visar på dina renar under de senaste 10 åren? innehåller något av dessa svarsalternativ

- Vet ej
- Ja

66) Vilka åtgärder sattes in när du senast såg renar drabbade av munröta/oral nekrobacillos/njunnevikke? Ange alla åtgärder som sattes in.

☐ Ingen åtgärd
☐ Gruppering av drabbade djur i sjukhägn
☐ Veterinär kontaktades
☐ Antibiotikabehandling
☐ Slakt
☐ Avlivning/kassering
☐ Behandling med övrigt läkemedel, ange vad i kommentarsfältet nedan
☐ Annat, ange vad i kommentarsfältet nedan

Denna informationsbox visas endast i läge förhandsgranskningen.

Följande kriterium måste vara uppfyllda för att följande fråga ska visas:

Om frågan Har du sett liknande förändringar som Bild 3. A-C visar på dina renar under de senaste 10 åren? innehåller något av dessa svarsalternativ

- Vet ej
- Ja

67) Plats för kommentarer:

Denna informationsbox visas endast i läge förhandsgranskningen.

Följande kriterium måste vara uppfyllda för att följande fråga ska visas:

Om frågan Har du sett liknande förändringar som Bild 3. A-C visar på dina renar under de senaste 10 åren? innehåller något av dessa svarsalternativ

- Vet ej
- Ja

Munröta/oral nekrobacillos/njunnevikke

68) Har utbrott eller fall av munröta/oral nekrobacillos/njunnevikke medfört ekonomiska konsekvenser?

- Ja
- Nej
- Vet ej

Denna informationsbox visas endast i läge förhandsgranskningen.

Följande kriterium måste vara uppfyllda för att följande fråga ska visas:

Om frågan Har du sett liknande förändringar som Bild 3. A-C visar på dina renar under de senaste 10 åren? innehåller något av dessa svarsalternativ

- Vet ej
- Ja

69) Plats för kommentarer:

Denna informationsbox visas endast i läge förhandsgranskningen.

Följande kriterium måste vara uppfyllda för att följande fråga ska visas:

Om frågan Har du sett liknande förändringar som Bild 3. A-C visar på dina renar under de senaste 10 åren? innehåller något av dessa svarsalternativ

- Vet ej
- Ja

Munröta/oral nekrobacillos/njunnevikke

Bild 3. A-C:

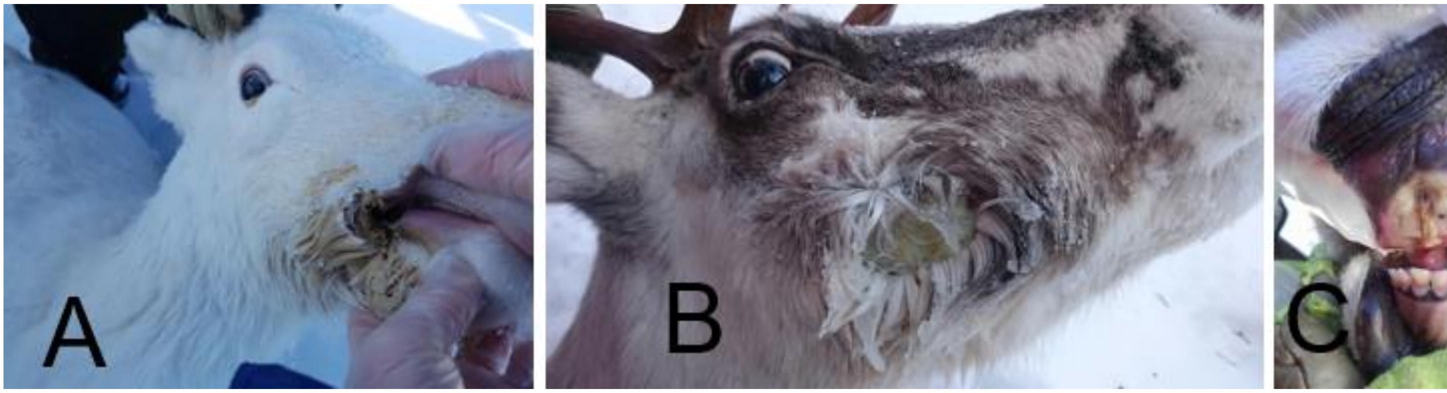

70) Har någon av förändringarna på bilderna ovan ett för dig eller för samebyn traditionellt namn?

- ○ Ja, ange i kommentarsfältet nedan
- ○ Nej
- ○ Vet ej

Denna informationsbox visas endast i läge förhandsgranskningen.

Följande kriterium måste vara uppfyllda för att följande fråga ska visas:

Om frågan Har du sett liknande förändringar som Bild 3. A-C visar på dina renar under de senaste 10 åren? innehåller något av dessa svarsalternativ

- Vet ej
- Ja

71) Plats för kommentarer/ange traditionellt namn:

Denna informationsbox visas endast i läge förhandsgranskningen.

Följande kriterium måste vara uppfyllda för att följande fråga ska visas:

Om frågan Har du sett liknande förändringar som Bild 3. A-C visar på dina renar under de senaste 10 åren? innehåller något av dessa svarsalternativ

- Vet ej
- Ja

72) Känner du till någon traditionell behandling av munröta/oral nekrobacillos/njunnevikke?

- ◯ Ja, beskriv i kommentarsfältet nedan
- ◯ Nej
- ◯ Vet ej

Denna informationsbox visas endast i läge förhandsgranskningen.

Följande kriterium måste vara uppfyllda för att följande fråga ska visas:

Om frågan Har du sett liknande förändringar som Bild 3. A-C visar på dina renar under de senaste 10 åren? innehåller något av dessa svarsalternativ

- Vet ej
- Ja

73) Plats för kommentarer:

Denna informationsbox visas endast i läge förhandsgranskningen.

Följande kriterium måste vara uppfyllda för att följande fråga ska visas:

Om frågan Har du sett liknande förändringar som Bild 3. A-C visar på dina renar under de senaste 10 åren? innehåller något av dessa svarsalternativ

- Vet ej
- Nej
- Ja

Nekrobacillos i magar på ren:

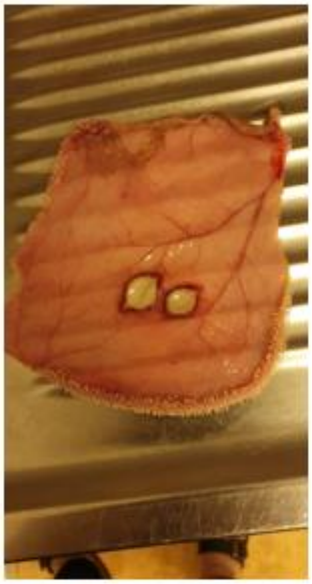

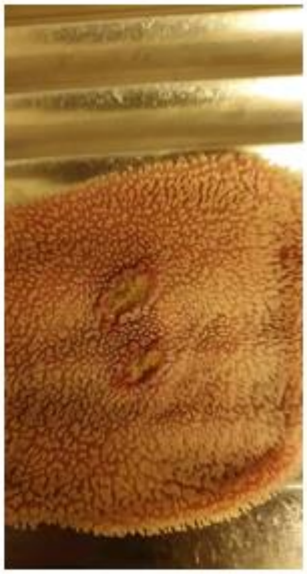

74) Har du sett liknande förändringar, som bilderna ovan visar, i magen på dina renar (vid slakt/obduktion)?

- ◯ Ja
- ◯ Nej
- ◯ Vet ej

Denna informationsbox visas endast i läge förhandsgranskningen.

Följande kriterium måste vara uppfyllda för att följande fråga ska visas:

Om frågan Har du sett liknande förändringar som Bild 3. A-C visar på dina renar under de senaste 10 åren? innehåller något av dessa svarsalternativ

- Vet ej
- Nej
- Ja

Strax följer frågor om övrig renhälsa

75) Lämna gärna övriga kommentarer kopplade till sjukdomen munröta/oral nekrobacillos/njunnevikke här:

4. Frågor om övriga sjukdomar och om renens hälsa:

76) * Vilka av följande sjukdomar har du observerat från vintersäsongen 2019/2020 fram till idag? Ange alla som observerats.

- ☐ Kastning/abort av foster
- ☐ Livmoderframfall
- ☐ Vinglighet
- ☐ Avmagring
- ☐ Blöt buk
- ☐ Diarré
- ☐ Skvalpmage
- ☐ Trumsjuka
- ☐ Hjärnhinnemask
- ☐ Parasiter (såsom hudkorm, svalgkorm, inälvsparasiter, hud/päls-parasiter)
- ☐ Självdöda djur utan orsak
- ☐ Annat, ange vad i kommentarer nedan.
- ☐ Ingen av ovan sjukdomar

77) Plats för kommentarer:

Denna informationsbox visas endast i läge förhandsgranskningen.

Följande kriterium måste vara uppfyllda för att följande fråga ska visas:

Om frågan Vilka av följande sjukdomar har du observerat från vintersäsongen 2019/2020 fram till idag? Ange alla som observerats. innehåller något av dessa svarsalternativ

- Blöt buk

78) Med vad utfodrades drabbade djur innan sjukdomen ’blöt buk’ bröt ut?

- Endast pellets
- Endast ensilage
- Kombination av pellets och ensilage
- Utfodrades ej
- Annat, ange i kommentarsfältet nedan.

Denna informationsbox visas endast i läge förhandsgranskningen.

Följande kriterium måste vara uppfyllda för att följande fråga ska visas:

Om frågan Vilka av följande sjukdomar har du observerat från vintersäsongen 2019/2020 fram till idag? Ange alla som observerats. innehåller något av dessa svarsalternativ

- Blöt buk

79) Plats för kommentarer:

Denna informationsbox visas endast i läge förhandsgranskningen.

Följande kriterium måste vara uppfyllda för att följande fråga ska visas:

Om frågan Vilka av följande sjukdomar har du observerat från vintersäsongen 2019/2020 fram till idag? Ange alla som observerats. innehåller något av dessa svarsalternativ

- Blöt buk

80) Har du observerat några andra/övriga symtom på renar som drabbats av blöt buk? Beskriv.

Denna informationsbox visas endast i läge förhandsgranskningen.

Följande kriterium måste vara uppfyllda för att följande fråga ska visas:

81) Får du den hjälp du har behov av från veterinär?

- Ja
- Nej, ange orsak i kommentarfältet nedan.

Denna informationsbox visas endast i läge förhandsgranskningen.

Följande kriterium måste vara uppfyllda för att följande fråga ska visas:

82) Plats för kommentarer:

83) Har du skickat iväg någon ren på obduktion?

- Ja
- Nej

84) Har du själv obducerat någon ren med veterinärhjälp?

- Ja
- Nej

85) Plats för kommentarer:

86) * Behandlas renarna regelbundet mot Korm?

- Ja
- Nej

Denna informationsbox visas endast i läge förhandsgranskningen.

Följande kriterium måste vara uppfyllda för att följande fråga ska visas:

Om frågan Behandlas renarna regelbundet mot Korm? innehåller något av dessa svarsalternativ

- Ja

87) Under vilken/vilka säsonger utförs vanligtvis behandlingen mot Korm?

☐ Vår
☐ Sommar
☐ Höst
☐ Vinter

Denna informationsbox visas endast i läge förhandsgranskningen.

Följande kriterium måste vara uppfyllda för att följande fråga ska visas:

Om frågan Behandlas renarna regelbundet mot Korm? innehåller något av dessa svarsalternativ

- Ja

88) Vilken grupp av djur behandlas vanligtvis mot Korm?

○ Endast livkalvar (1 år eller yngre)
○ Endast vajor (över 1 år)
○ Endast handjur (över 1 år)
○ Kombination livkalvar, handjur eller vajor
○ Annat, ange vad i kommentarsfältet nedan.

Denna informationsbox visas endast i läge förhandsgranskningen.

Följande kriterium måste vara uppfyllda för att följande fråga ska visas:

Om frågan Behandlas renarna regelbundet mot Korm? innehåller något av dessa svarsalternativ

- Ja

89) Plats för kommentarer:

Bild 4. A-B. Vanlig fästing

Ixodes ricinus

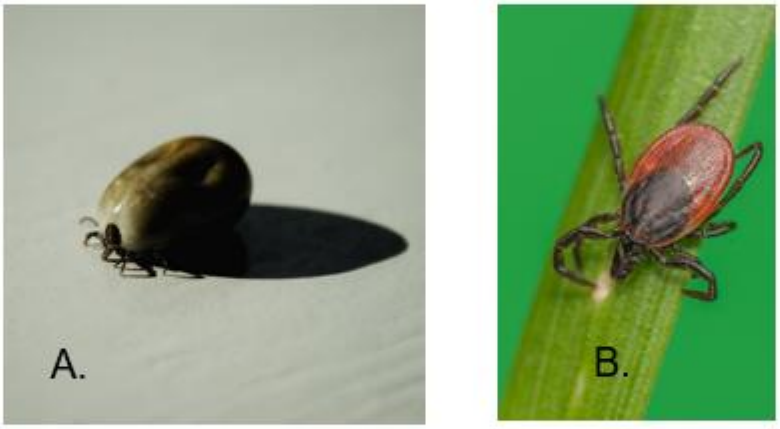

Foto: Johan Werner Foto: Anders Lindström.

A: Blodfylld hona.

B: Hona på ett grässtrå.

90) Fästingar kan sprida smittsamma sjukdomar och på grund av det allt mildare klimatet sprider sig fästingar norrut. Har du sett fästingar på dina renar (se bild 4. A-B ovan)?

- ◯ Ja
- ◯ Nej
- ◯ Vet ej

Strax följer frågor om utfodring

91) Lämna gärna övriga kommentarer kopplade till renhälsa här:

5. Nu har du bara frågor om utfodring kvar innan enkäten är klar!

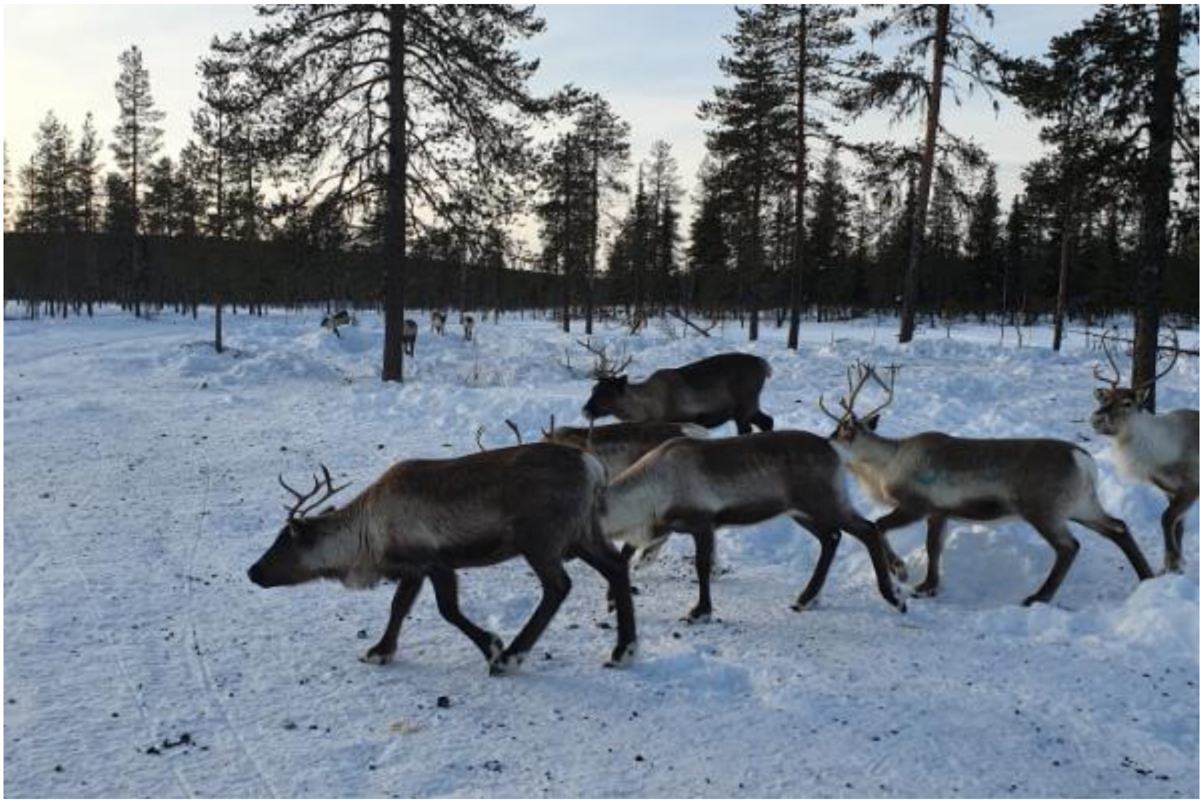

Frågor om utfodring är uppdelade enligt följande:

1. Allmän del

2. Effekter av utfodring

3. Utfodringsrutiner (gäller under 2019/2020)

4. Övrigt (gäller senaste fem åren)

92) * Har du utfodrat (nöd- eller stödutfodrat) dina renar i din vintergruppen vid något tillfälle under de senaste fem åren? Om samebyn inte är uppdelad i olika vintergrupper svarar du för hela samebyn. Obs: här avses utfodring av vintergrupp (slakt- och livren) i hägn och/eller på fribete i över två veckor, men ej under flytt och samling som pågår under en kortare period än två veckor.

- ○ Ja
- ○ Nej

Denna informationsbox visas endast i läge förhandsgranskningen.

Följande kriterium måste vara uppfyllda för att följande fråga ska visas:

Om frågan Har du utfodrat (nöd- eller stödutfodrat) dina renar i din vintergruppen vid något tillfälle under de senaste fem åren? Om samebyn inte är uppdelad i olika vintergrupper svarar du för hela samebyn. Obs: här avses utfodring av vintergrupp (slakt- och livren) i hägn och/eller på fribete i över två veckor, men ej under flytt och samling som pågår under en kortare period än två veckor. innehåller något av dessa svarsalternativ

- Ja

UTFODRING

1. Allmän del

Här avses utfodring (nöd- eller stödutfodring) av dina renar (slakt- och livrenar) i vintergruppen i över två veckor, i hägn eller på fribete, men ej under flytt och samling som pågår under en kortare period än två veckor. Om vintergruppen inte är uppdelad svarar du för hela samebyn.

93) * Ange under var och en av följande säsonger, var renarna utfodrades alternativt att de inte utfodrades alls. Svara för varje säsong, ett/flera alternativ.

	Ej utfodrade	Utfodrade i hägn	Utfodrade på fribete	Vet ej
2015/2016	☐	☐	☐	☐
2016/2017	☐	☐	☐	☐
2017/2018	☐	☐	☐	☐
2018/2019	☐	☐	☐	☐
2019/2020	☐	☐	☐	☐
2020/2021	☐	☐	☐	☐

Denna informationsbox visas endast i läge förhandsgranskningen.

Följande kriterium måste vara uppfyllda för att följande fråga ska visas:

Om frågan Har du utfodrat (nöd- eller stödutfodrat) dina renar i din vintergruppen vid något tillfälle under de senaste fem åren? Om samebyn inte är uppdelad i olika

vintergrupper svarar du för hela samebyn. Obs: här avses utfodring av vintergrupp (slakt- och livren) i hägn och/eller på fribete i över två veckor, men ej under flytt och samling som pågår under en kortare period än två veckor. innehåller något av dessa svarsalternativ

- Ja

94) Plats för kommentarer:

Denna informationsbox visas endast i läge förhandsgranskningen.

Följande kriterium måste vara uppfyllda för att följande fråga ska visas:

Om frågan Har du utfodrat (nöd- eller stödutfodrat) dina renar i din vintergruppen vid något tillfälle under de senaste fem åren? Om samebyn inte är uppdelad i olika vintergrupper svarar du för hela samebyn. Obs: här avses utfodring av vintergrupp (slakt- och livren) i hägn och/eller på fribete i över två veckor, men ej under flytt och samling som pågår under en kortare period än två veckor. innehåller något av dessa svarsalternativ

- Ja

UTFODRING

1. Allmän del

Avser utfodring (nöd- eller stödutfodring) av dina renar (slakt och livrenar) i vintergruppen i över två veckor, i hägn eller på fribete, men ej under flytt och samling som pågår under en kortare period än två veckor. Om vintergruppen inte är uppdelad svarar du för hela samebyn.

95) Varför vinterutfodrades renarna under de senaste fem åren? Ange alla alternativ som stämmer för livren respektive för slaktren. Kommentera eventuella skillnader mellan åren i kommentarsfältet nedan.

	Livren	Slaktren
Dåliga vinterbetesförhållanden	☐	☐
Överlevnad	☐	☐
Bättre tillväxt	☐	☐

	Livren	Slaktren
Rovdjur	☐	☐
Sjukdom/dåligt hull (sjukhägn)	☐	☐
Minska cesiumhalt (Tjernobylolyckan)	☐	☐
Konkurrerande markanvändning, utveckla gärna i kommentarsfältet nedan	☐	☐
Annat, ange i kommentarsfältet nedan.	☐	☐

Denna informationsbox visas endast i läge förhandsgranskningen.

Följande kriterium måste vara uppfyllda för att följande fråga ska visas:

Om frågan Har du utfodrat (nöd- eller stödutfodrat) dina renar i din vintergruppen vid något tillfälle under de senaste fem åren? Om samebyn inte är uppdelad i olika vintergrupper svarar du för hela samebyn. Obs: här avses utfodring av vintergrupp (slakt- och livren) i hägn och/eller på fribete i över två veckor, men ej under flytt och samling som pågår under en kortare period än två veckor. innehåller något av dessa svarsalternativ

- Ja

96) Plats för kommentarer, ange om det gäller för livren eller slaktren:

Denna informationsbox visas endast i läge förhandsgranskningen.

Följande kriterium måste vara uppfyllda för att följande fråga ska visas:

Om frågan Har du utfodrat (nöd- eller stödutfodrat) dina renar i din vintergruppen vid något tillfälle under de senaste fem åren? Om samebyn inte är uppdelad i olika vintergrupper svarar du för hela samebyn. Obs: här avses utfodring av vintergrupp (slakt- och livren) i hägn och/eller på fribete i över två veckor, men ej under flytt och samling som pågår under en kortare period än två veckor. innehåller något av dessa svarsalternativ

- Ja

2. Effekter av utfodring

97) Upplever du en förändring i beteendet hos renar som utfodrats under en sammanhängande period på minst två veckor när de åter släppts på fribete? (Exempelvis tamhetsgrad, enklare eller svårare att samla, obs: avser ej beteendeförändring under utfodringen.)

	Ja	Nej	Vet ej
Bland renar utfodrade i hägn	○	○	○
Bland renar utfodrade på fribete	○	○	○

Denna informationsbox visas endast i läge förhandsgranskningen.

Följande kriterium måste vara uppfyllda för att följande fråga ska visas:

Om frågan Har du utfodrat (nöd- eller stödutfodrat) dina renar i din vintergruppen vid något tillfälle under de senaste fem åren? Om samebyn inte är uppdelad i olika vintergrupper svarar du för hela samebyn. Obs: här avses utfodring av vintergrupp (slakt- och livren) i hägn och/eller på fribete i över två veckor, men ej under flytt och samling som pågår under en kortare period än två veckor. innehåller något av dessa svarsalternativ

- Ja

98) Plats för kommentarer:

Denna informationsbox visas endast i läge förhandsgranskningen.

Följande kriterium måste vara uppfyllda för att följande fråga ska visas:

Om frågan Har du utfodrat (nöd- eller stödutfodrat) dina renar i din vintergruppen vid något tillfälle under de senaste fem åren? Om samebyn inte är uppdelad i olika vintergrupper svarar du för hela samebyn. Obs: här avses utfodring av vintergrupp (slakt- och livren) i hägn och/eller på fribete i över två veckor, men ej under flytt och

samling som pågår under en kortare period än två veckor. innehåller något av dessa svarsalternativ

- Ja

2. Effekter av utfodring

99) Upplever du att kalvar som utfodrats under vintern har högre slaktvikt och hull kommande höst jämfört med ej utfodrade kalvar?

- Ja
- Nej
- Vet ej

Denna informationsbox visas endast i läge förhandsgranskningen.

Följande kriterium måste vara uppfyllda för att följande fråga ska visas:

Om frågan Har du utfodrat (nöd- eller stödutfodrat) dina renar i din vintergruppen vid något tillfälle under de senaste fem åren? Om samebyn inte är uppdelad i olika vintergrupper svarar du för hela samebyn. Obs: här avses utfodring av vintergrupp (slakt- och livren) i hägn och/eller på fribete i över två veckor, men ej under flytt och samling som pågår under en kortare period än två veckor. innehåller något av dessa svarsalternativ

- Ja

100) Upplever du att kalvfrekvensen (andel vajor med kalv) vid kalvmärkning och/eller på hösten påverkas positivt av utfodring av vajor vintern innan?

- Ja
- Nej
- Vet ej

Denna informationsbox visas endast i läge förhandsgranskningen.

Följande kriterium måste vara uppfyllda för att följande fråga ska visas:

Om frågan Har du utfodrat (nöd- eller stödutfodrat) dina renar i din vintergruppen vid något tillfälle under de senaste fem åren? Om samebyn inte är uppdelad i olika vintergrupper svarar du för hela samebyn. Obs: här avses utfodring av vintergrupp (slakt- och livren) i hägn och/eller på fribete i över två veckor, men ej under flytt och

samling som pågår under en kortare period än två veckor. innehåller något av dessa svarsalternativ

- Ja

101) Plats för kommentarer:

Denna informationsbox visas endast i läge förhandsgranskningen.

Följande kriterium måste vara uppfyllda för att följande fråga ska visas:

Om frågan Har du utfodrat (nöd- eller stödutfodrat) dina renar i din vintergruppen vid något tillfälle under de senaste fem åren? Om samebyn inte är uppdelad i olika vintergrupper svarar du för hela samebyn. Obs: här avses utfodring av vintergrupp (slakt- och livren) i hägn och/eller på fribete i över två veckor, men ej under flytt och samling som pågår under en kortare period än två veckor. innehåller något av dessa svarsalternativ

- Ja

2. Effekter av utfodring

102) Upplever du att utfodring påverkar kalvarnas förmåga att söka och hitta bete efterföljande vinter?

- ○ Ja
- ○ Nej
- ○ Vet ej

Denna informationsbox visas endast i läge förhandsgranskningen.

Följande kriterium måste vara uppfyllda för att följande fråga ska visas:

Om frågan Har du utfodrat (nöd- eller stödutfodrat) dina renar i din vintergruppen vid något tillfälle under de senaste fem åren? Om samebyn inte är uppdelad i olika vintergrupper svarar du för hela samebyn. Obs: här avses utfodring av vintergrupp (slakt- och livren) i hägn och/eller på fribete i över två veckor, men ej under flytt och

samling som pågår under en kortare period än två veckor. innehåller något av dessa svarsalternativ

- Ja

103) Plats för kommentarer:

Denna informationsbox visas endast i läge förhandsgranskningen.

Följande kriterium måste vara uppfyllda för att följande fråga ska visas:

Om frågan Har du utfodrat (nöd- eller stödutfodrat) dina renar i din vintergruppen vid något tillfälle under de senaste fem åren? Om samebyn inte är uppdelad i olika vintergrupper svarar du för hela samebyn. Obs: här avses utfodring av vintergrupp (slakt- och livren) i hägn och/eller på fribete i över två veckor, men ej under flytt och samling som pågår under en kortare period än två veckor. innehåller något av dessa svarsalternativ

- Ja

och

Om frågan 2019/2020 innehåller något av dessa svarsalternativ

- Utfodrade på fribete
- Utfodrade i hägn

3. Frågor kopplade till utfodringsrutiner under vinter- och/eller vårsäsongen i din vintergrupp, 2019/2020.

Avser fortsatt utfodring (nöd- eller stödutfodring) av dina renar i vintergruppen i över två veckor, men ej under flytt och samling under en kortare period än två veckor. Om vintergruppen inte är uppdelad svarar du för hela samebyn.

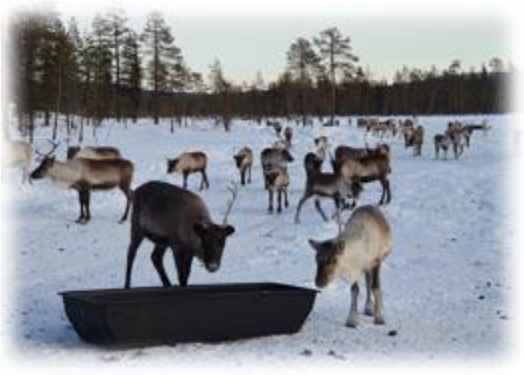

104) Ungefär hur många renar utfodrades totalt i din vintergrupp säsongen 2019/2020?

	I hägn	På fribete
Under 100	☐	☐
100-299	☐	☐
300-499	☐	☐
500-999	☐	☐
Över 1000	☐	☐

Denna informationsbox visas endast i läge förhandsgranskningen.

Följande kriterium måste vara uppfyllda för att följande fråga ska visas:

Om frågan Har du utfodrat (nöd- eller stödutfodrat) dina renar i din vintergruppen vid något tillfälle under de senaste fem åren? Om samebyn inte är uppdelad i olika vintergrupper svarar du för hela samebyn. Obs: här avses utfodring av vintergrupp (slakt- och livren) i hägn och/eller på fribete i över två veckor, men ej under flytt och samling som pågår under en kortare period än två veckor. innehåller något av dessa svarsalternativ

- Ja

och

Om frågan 2019/2020 innehåller något av dessa svarsalternativ

- Utfodrade på fribete
- Utfodrade i hägn

105) Vilken/vilka grupper av djur utfodrades i huvudsak av följande tre kategorier, och var befanns renarna då? Välj ett/flera alternativ.

	I hägn	På fribete
Hela vintergruppen	☐	☐
I huvudsak kalvar (1 år eller yngre)	☐	☐
I huvudsak vuxna djur (över 1 år)	☐	☐

Denna informationsbox visas endast i läge förhandsgranskningen.

Följande kriterium måste vara uppfyllda för att följande fråga ska visas:

Om frågan Har du utfodrat (nöd- eller stödutfodrat) dina renar i din vintergruppen vid något tillfälle under de senaste fem åren? Om samebyn inte är uppdelad i olika vintergrupper svarar du för hela samebyn. Obs: här avses utfodring av vintergrupp (slakt- och livren) i hägn och/eller på fribete i över två veckor, men ej under flytt och samling som pågår under en kortare period än två veckor. innehåller något av dessa svarsalternativ

- Ja

106) Plats för kommentarer:

Denna informationsbox visas endast i läge förhandsgranskningen.

Följande kriterium måste vara uppfyllda för att följande fråga ska visas:

Om frågan Har du utfodrat (nöd- eller stödutfodrat) dina renar i din vintergruppen vid något tillfälle under de senaste fem åren? Om samebyn inte är uppdelad i olika vintergrupper svarar du för hela samebyn. Obs: här avses utfodring av vintergrupp (slakt- och livren) i hägn och/eller på fribete i över två veckor, men ej under flytt och samling som pågår under en kortare period än två veckor. innehåller något av dessa svarsalternativ

- Ja

och

Om frågan 2019/2020 innehåller något av dessa svarsalternativ

- Utfodrade på fribete

- Utfodrade i hägn

3. Frågor kopplade till utfodringsrutiner under vinter- och/eller vårsäsongen i din vintergrupp, 2019/2020.

Frågorna avser att kartlägga rutiner vid utfodring i dagsläget.

107) Ungefär under hur lång tidsperiod utfodrade du dina renar sammanhängande (ange från att den första renen började utfodras)? Avser stöd eller nödutfodring över två veckors tid under 2019/2020.

	Mindre än 1 månad	1-3 månader	Över 3 månader
Utfodring i hägn	◯	◯	◯
Utfodring på fribete	◯	◯	◯

Denna informationsbox visas endast i läge förhandsgranskningen.

Följande kriterium måste vara uppfyllda för att följande fråga ska visas:

Om frågan Har du utfodrat (nöd- eller stödutfodrat) dina renar i din vintergruppen vid något tillfälle under de senaste fem åren? Om samebyn inte är uppdelad i olika vintergrupper svarar du för hela samebyn. Obs: här avses utfodring av vintergrupp (slakt- och livren) i hägn och/eller på fribete i över två veckor, men ej under flytt och samling som pågår under en kortare period än två veckor. innehåller något av dessa svarsalternativ

- Ja

och

Om frågan 2019/2020 innehåller något av dessa svarsalternativ

- Utfodrade på fribete
- Utfodrade i hägn

108) Plats för kommentarer:

Denna informationsbox visas endast i läge förhandsgranskningen.

Följande kriterium måste vara uppfyllda för att följande fråga ska visas:

Om frågan Har du utfodrat (nöd- eller stödutfodrat) dina renar i din vintergruppen vid något tillfälle under de senaste fem åren? Om samebyn inte är uppdelad i olika vintergrupper svarar du för hela samebyn. Obs: här avses utfodring av vintergrupp (slakt- och livren) i hägn och/eller på fribete i över två veckor, men ej under flytt och samling som pågår under en kortare period än två veckor. innehåller något av dessa svarsalternativ

- Ja

och

Om frågan 2019/2020 innehåller något av dessa svarsalternativ

- Utfodrade i hägn

3. Frågor kopplade till utfodringsrutiner under vinter- och/eller vårsäsongen i din vintergrupp, 2019/2020.

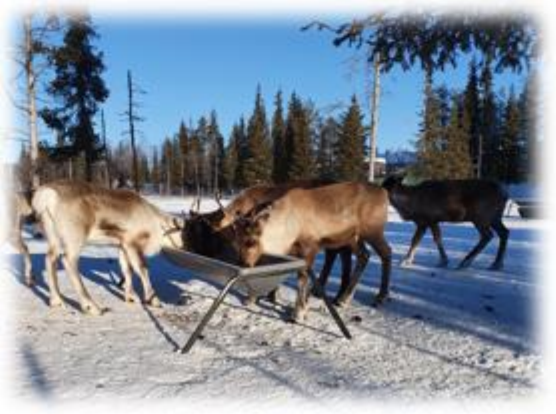

Frågorna avser att kartlägga rutiner vid utfodring i dagsläget.

109) * För att kartlägga rutiner och förutsättningar inom renskötseln följer frågor om olika fodermedel. Vilka av följande fodermedel utfodrade du dina renar med i hägn 2019/2020?

- ◯ Endast grovfoder (ensilage/hösilage/hö)
- ◯ Endast pellets
- ◯ Kombination av grovfoder och pellets

Denna informationsbox visas endast i läge förhandsgranskningen.

Följande kriterium måste vara uppfyllda för att följande fråga ska visas:

Om frågan Har du utfodrat (nöd- eller stödutfodrat) dina renar i din vintergruppen vid något tillfälle under de senaste fem åren? Om samebyn inte är uppdelad i olika vintergrupper svarar du för hela samebyn. Obs: här avses utfodring av vintergrupp (slakt- och livren) i hägn och/eller på fribete i över två veckor, men ej under flytt och samling som pågår under en kortare period än två veckor. innehåller något av dessa svarsalternativ

- Ja

och

Om frågan 2019/2020 innehåller något av dessa svarsalternativ

- Utfodrade på fribete

110) * Vilka av följande fodermedel utfodrade du dina renar med på fribete 2019/2020?

- Endast grovfoder (ensilage/hösilage/hö)
- Endast pellets
- Kombination av grovfoder och pellets

Denna informationsbox visas endast i läge förhandsgranskningen.

Följande kriterium måste vara uppfyllda för att följande fråga ska visas:

Om frågan Har du utfodrat (nöd- eller stödutfodrat) dina renar i din vintergruppen vid något tillfälle under de senaste fem åren? Om samebyn inte är uppdelad i olika vintergrupper svarar du för hela samebyn. Obs: här avses utfodring av vintergrupp (slakt- och livren) i hägn och/eller på fribete i över två veckor, men ej under flytt och samling som pågår under en kortare period än två veckor. innehåller något av dessa svarsalternativ

- Ja

och

Om frågan 2019/2020 innehåller något av dessa svarsalternativ

- Utfodrade på fribete
- Utfodrade i hägn

111) Plats för kommentarer:

Denna informationsbox visas endast i läge förhandsgranskningen.

Följande kriterium måste vara uppfyllda för att följande fråga ska visas:

Om frågan Har du utfodrat (nöd- eller stödutfodrat) dina renar i din vintergruppen vid något tillfälle under de senaste fem åren? Om samebyn inte är uppdelad i olika vintergrupper svarar du för hela samebyn. Obs: här avses utfodring av vintergrupp (slakt- och livren) i hägn och/eller på fribete i över två veckor, men ej under flytt och samling som pågår under en kortare period än två veckor. innehåller något av dessa svarsalternativ

- Ja

och

Om frågan 2019/2020 innehåller något av dessa svarsalternativ

- Utfodrade på fribete
- Utfodrade i hägn

3. Frågor kopplade till utfodringsrutiner under vinter- och/eller vårsäsongen i din vintergrupp, 2019/2020.

112) När utfodrade du med ren- och/eller hänglav utöver det som fanns tillgängligt naturlig 2019/2020? Ange ett/flera val.

	I hägn	På fribete
Som tillskott vid utfodring	☐	☐
Till sjuka/svaga renar	☐	☐
Vid tillvänjning av foder	☐	☐
Har ej förutsättningar/tillgång till lav	☐	☐
Annat, ange i kommentarer nedan	☐	☐

Denna informationsbox visas endast i läge förhandsgranskningen.

Följande kriterium måste vara uppfyllda för att följande fråga ska visas:

Om frågan Har du utfodrat (nöd- eller stödutfodrat) dina renar i din vintergruppen vid något tillfälle under de senaste fem åren? Om samebyn inte är uppdelad i olika vintergrupper svarar du för hela samebyn. Obs: här avses utfodring av vintergrupp (slakt- och livren) i hägn och/eller på fribete i över två veckor, men ej under flytt och samling som pågår under en kortare period än två veckor. innehåller något av dessa svarsalternativ

- Ja

och

Om frågan 2019/2020 innehåller något av dessa svarsalternativ

- Utfodrade på fribete
- Utfodrade i hägn

och

Om frågan För att kartlägga rutiner och förutsättningar inom renskötseln följer frågor om olika fodermedel. Vilka av följande fodermedel utfodrade du dina renar med i hägn 2019/2020? innehåller något av dessa svarsalternativ

- Kombination av grovfoder och pellets
- Endast grovfoder (ensilage/hösilage/hö)

och

Om frågan Vilka av följande fodermedel utfodrade du dina renar med på fribete 2019/2020? innehåller något av dessa svarsalternativ

- Kombination av grovfoder och pellets
- Endast grovfoder (ensilage/hösilage/hö)

113) Om möjligt, uppskatta antal grovfoder/ensilagebalar och ange typ (exempel rund,- eller fyrkantsbal) som användes under utfodringsperioden 2019/2020?

I hägn

På fribete

Denna informationsbox visas endast i läge förhandsgranskningen.

Följande kriterium måste vara uppfyllda för att följande fråga ska visas:

Om frågan Har du utfodrat (nöd- eller stödutfodrat) dina renar i din vintergruppen vid något tillfälle under de senaste fem åren? Om samebyn inte är uppdelad i olika vintergrupper svarar du för hela samebyn. Obs: här avses utfodring av vintergrupp (slakt- och livren) i hägn och/eller på fribete i över två veckor, men ej under flytt och

samling som pågår under en kortare period än två veckor. innehåller något av dessa svarsalternativ

- Ja

och

Om frågan 2019/2020 innehåller något av dessa svarsalternativ

- Utfodrade på fribete
- Utfodrade i hägn

114) Plats för kommentarer:

Denna informationsbox visas endast i läge förhandsgranskningen.

Följande kriterium måste vara uppfyllda för att följande fråga ska visas:

Om frågan Har du utfodrat (nöd- eller stödutfodrat) dina renar i din vintergruppen vid något tillfälle under de senaste fem åren? Om samebyn inte är uppdelad i olika vintergrupper svarar du för hela samebyn. Obs: här avses utfodring av vintergrupp (slakt- och livren) i hägn och/eller på fribete i över två veckor, men ej under flytt och samling som pågår under en kortare period än två veckor. innehåller något av dessa svarsalternativ

- Ja

och

Om frågan 2019/2020 innehåller något av dessa svarsalternativ

- Utfodrade på fribete
- Utfodrade i hägn

och

Om frågan För att kartlägga rutiner och förutsättningar inom renskötseln följer frågor om olika fodermedel. Vilka av följande fodermedel utfodrade du dina renar med i hägn 2019/2020? innehåller något av dessa svarsalternativ

- Kombination av grovfoder och pellets
- Endast pellets

och

Om frågan Vilka av följande fodermedel utfodrade du dina renar med på fribete 2019/2020? innehåller något av dessa svarsalternativ

- Kombination av grovfoder och pellets
- Endast pellets

3. Frågor kopplade till utfodringsrutiner under vinter- och/eller vårsäsongen i din vintergrupp, 2019/2020.

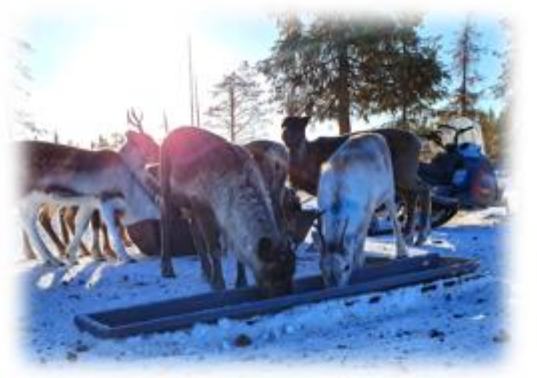

115) Ange antalet utfodringstillfällen per dag med pellets (2019/2020).

	1	2 eller flera
I hägn	○	○
På fribete	○	○

Denna informationsbox visas endast i läge förhandsgranskningen.

Följande kriterium måste vara uppfyllda för att följande fråga ska visas:

Om frågan Har du utfodrat (nöd- eller stödutfodrat) dina renar i din vintergruppen vid något tillfälle under de senaste fem åren? Om samebyn inte är uppdelad i olika vintergrupper svarar du för hela samebyn. Obs: här avses utfodring av vintergrupp (slakt- och livren) i hägn och/eller på fribete i över två veckor, men ej under flytt och samling som pågår under en kortare period än två veckor. innehåller något av dessa svarsalternativ

- Ja

och

Om frågan 2019/2020 innehåller något av dessa svarsalternativ

- Utfodrade på fribete
- Utfodrade i hägn

och

Om frågan För att kartlägga rutiner och förutsättningar inom renskötseln följer frågor om olika fodermedel. Vilka av följande fodermedel utfodrade du dina renar med i hägn 2019/2020? innehåller något av dessa svarsalternativ

- Kombination av grovfoder och pellets
- Endast pellets

och

Om frågan Vilka av följande fodermedel utfodrade du dina renar med på fribete 2019/2020? innehåller något av dessa svarsalternativ

- Kombination av grovfoder och pellets
- Endast pellets

116) Om möjligt, uppskatta antal kilo pellets per djur och dag i genomsnitt för säsongen 2019/2020 och ange om det gäller för nöd,- och eller stödutfodring.

	Nödutfodring	Stödutfodring
I hägn		
På fribete		

Denna informationsbox visas endast i läge förhandsgranskningen.

Följande kriterium måste vara uppfyllda för att följande fråga ska visas:

Om frågan Har du utfodrat (nöd- eller stödutfodrat) dina renar i din vintergruppen vid något tillfälle under de senaste fem åren? Om samebyn inte är uppdelad i olika vintergrupper svarar du för hela samebyn. Obs: här avses utfodring av vintergrupp (slakt- och livren) i hägn och/eller på fribete i över två veckor, men ej under flytt och samling som pågår under en kortare period än två veckor. innehåller något av dessa svarsalternativ

- Ja

och

Om frågan 2019/2020 innehåller något av dessa svarsalternativ

- Utfodrade på fribete
- Utfodrade i hägn

och

Om frågan För att kartlägga rutiner och förutsättningar inom renskötseln följer frågor om olika fodermedel. Vilka av följande fodermedel utfodrade du dina renar med i hägn 2019/2020? innehåller något av dessa svarsalternativ

- Kombination av grovfoder och pellets
- Endast pellets

och

Om frågan Vilka av följande fodermedel utfodrade du dina renar med på fribete 2019/2020? innehåller något av dessa svarsalternativ

- Kombination av grovfoder och pellets
- Endast pellets

117) Plats för kommentarer:

Denna informationsbox visas endast i läge förhandsgranskningen.

Följande kriterium måste vara uppfyllda för att följande fråga ska visas:

Om frågan Har du utfodrat (nöd- eller stödutfodrat) dina renar i din vintergruppen vid något tillfälle under de senaste fem åren? Om samebyn inte är uppdelad i olika vintergrupper svarar du för hela samebyn. Obs: här avses utfodring av vintergrupp (slakt- och livren) i hägn och/eller på fribete i över två veckor, men ej under flytt och samling som pågår under en kortare period än två veckor. innehåller något av dessa svarsalternativ

- Ja

och

Om frågan 2019/2020 innehåller något av dessa svarsalternativ

- Utfodrade på fribete
- Utfodrade i hägn

och

Om frågan För att kartlägga rutiner och förutsättningar inom renskötseln följer frågor om olika fodermedel. Vilka av följande fodermedel utfodrade du dina renar med i hägn 2019/2020? innehåller något av dessa svarsalternativ

- Kombination av grovfoder och pellets
- Endast pellets

och

Om frågan Vilka av följande fodermedel utfodrade du dina renar med på fribete 2019/2020? innehåller något av dessa svarsalternativ

- Kombination av grovfoder och pellets
- Endast pellets

3. Frågor kopplade till utfodringsrutiner under vinter- och/eller vårsäsongen i din vintergrupp, 2019/2020.

118) Om möjligt, ange foderleverantör av pellets under 2019/2020.

I hägn:

På fribete:

Denna informationsbox visas endast i läge förhandsgranskningen.

Följande kriterium måste vara uppfyllda för att följande fråga ska visas:

Om frågan Har du utfodrat (nöd- eller stödutfodrat) dina renar i din vintergruppen vid något tillfälle under de senaste fem åren? Om samebyn inte är uppdelad i olika vintergrupper svarar du för hela samebyn. Obs: här avses utfodring av vintergrupp (slakt- och livren) i hägn och/eller på fribete i över två veckor, men ej under flytt och samling som pågår under en kortare period än två veckor. innehåller något av dessa svarsalternativ

- Ja

och

Om frågan 2019/2020 innehåller något av dessa svarsalternativ

- Utfodrade på fribete
- Utfodrade i hägn

och

Om frågan För att kartlägga rutiner och förutsättningar inom renskötseln följer frågor om olika fodermedel. Vilka av följande fodermedel utfodrade du dina renar med i hägn 2019/2020? innehåller något av dessa svarsalternativ

- Kombination av grovfoder och pellets
- Endast pellets

och

Om frågan Vilka av följande fodermedel utfodrade du dina renar med på fribete 2019/2020? innehåller något av dessa svarsalternativ

- Kombination av grovfoder och pellets
- Endast pellets

119) Hur utfodrades vanligtvis pelletsen under föregående säsong (2019/2020)? Kryssa i ett/flera alternativ.

	I hägn	På fribete
Direkt på marken	☐	☐
Krubba på ben	☐	☐
Krubba utan ben	☐	☐
Annat, ange i kommenarsfältet nedan.	☐	☐

Denna informationsbox visas endast i läge förhandsgranskningen.

Följande kriterium måste vara uppfyllda för att följande fråga ska visas:

Om frågan Har du utfodrat (nöd- eller stödutfodrat) dina renar i din vintergruppen vid något tillfälle under de senaste fem åren? Om samebyn inte är uppdelad i olika vintergrupper svarar du för hela samebyn. Obs: här avses utfodring av vintergrupp (slakt- och livren) i hägn och/eller på fribete i över två veckor, men ej under flytt och samling som pågår under en kortare period än två veckor. innehåller något av dessa svarsalternativ

- Ja

och

Om frågan 2019/2020 innehåller något av dessa svarsalternativ

- Utfodrade på fribete
- Utfodrade i hägn

och

Om frågan För att kartlägga rutiner och förutsättningar inom renskötseln följer frågor om olika fodermedel. Vilka av följande fodermedel utfodrade du dina renar med i hägn 2019/2020? innehåller något av dessa svarsalternativ

- Kombination av grovfoder och pellets
- Endast pellets

och

Om frågan Vilka av följande fodermedel utfodrade du dina renar med på fribete 2019/2020? innehåller något av dessa svarsalternativ

- Kombination av grovfoder och pellets
- Endast pellets

120) Plats för kommentarer:

Denna informationsbox visas endast i läge förhandsgranskningen.

Följande kriterium måste vara uppfyllda för att följande fråga ska visas:

Om frågan Har du utfodrat (nöd- eller stödutfodrat) dina renar i din vintergruppen vid något tillfälle under de senaste fem åren? Om samebyn inte är uppdelad i olika vintergrupper svarar du för hela samebyn. Obs: här avses utfodring av vintergrupp (slakt- och livren) i hägn och/eller på fribete i över två veckor, men ej under flytt och samling som pågår under en kortare period än två veckor. innehåller något av dessa svarsalternativ

- Ja

4. Frågor om övriga fodermedel och rutiner vid utfodring under de senaste fem åren.

Avser fortsatt utfodring (nöd- eller stödutfodring) av dina renar (slakt- och livrenar) i vintergruppen i över två veckor, men ej under flytt och samling under en kortare period än två veckor. Om vintergruppen inte är uppdelad svarar du för hela samebyn.

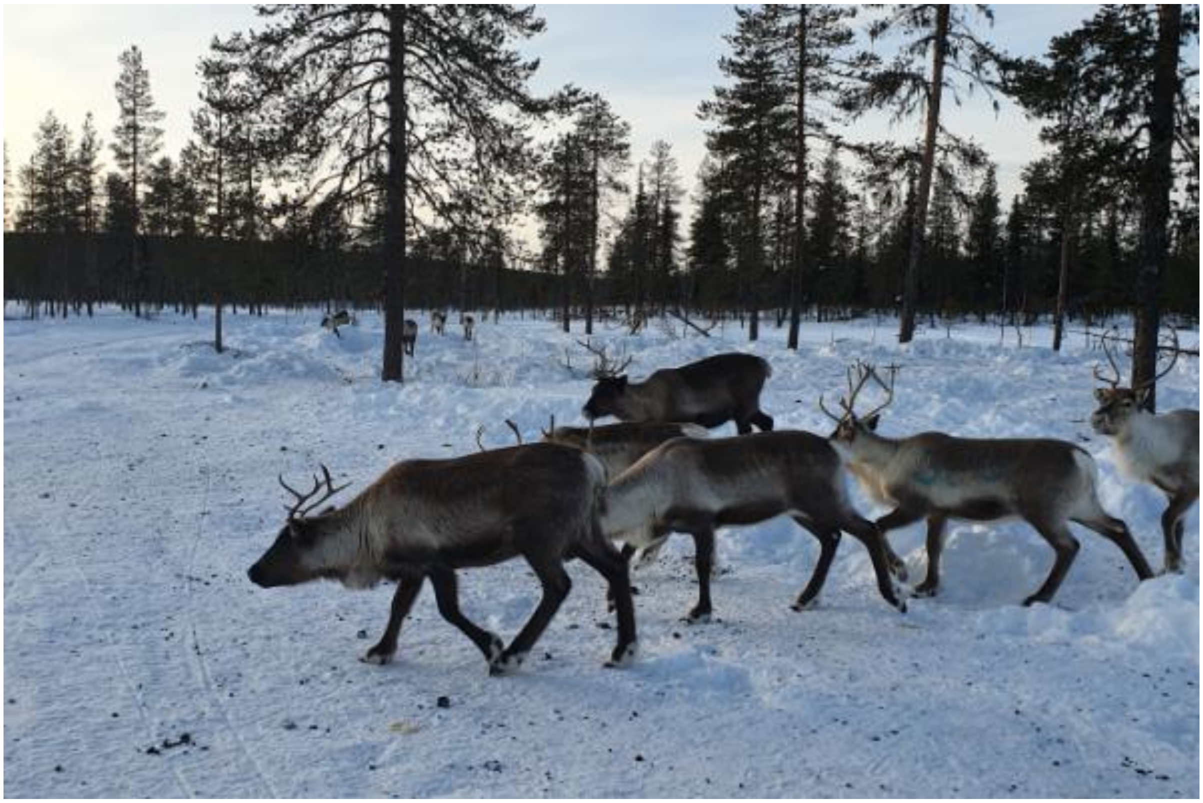

Frågorna avser att kartlägga rutiner vid utfodring i dagsläget.

121) Hur vanligt förekommande är det att renarna ges tillgång till mineraltillskott (exempelvis mineralsaltsten, mineralbalja, foderjäst, injektion) vid utfodring under de senaste fem åren? Välj ett/flera alternativ.

	I hägn	På fribete
I princip alltid vid utfodring (anges som enda svarsalternativ)	☐	☐
Periodvis vid utfodring under säsongen	☐	☐
Enstaka säsonger	☐	☐
Aldrig (anges som enda svarsalternativ)	☐	☐
Under särskilda omständigheter (exempelvis till renar i sämre kondition och i sjukhägn)	☐	☐
Annat, ange i kommentarer nedan	☐	☐

Denna informationsbox visas endast i läge förhandsgranskningen.

Följande kriterium måste vara uppfyllda för att följande fråga ska visas:

Om frågan Har du utfodrat (nöd- eller stödutfodrat) dina renar i din vintergruppen vid något tillfälle under de senaste fem åren? Om samebyn inte är uppdelad i olika vintergrupper svarar du för hela samebyn. Obs: här avses utfodring av vintergrupp (slakt- och livren) i hägn och/eller på fribete i över två veckor, men ej under flytt och samling som pågår under en kortare period än två veckor. innehåller något av dessa svarsalternativ

- Ja

122) Plats för kommentarer:

Denna informationsbox visas endast i läge förhandsgranskningen.

Följande kriterium måste vara uppfyllda för att följande fråga ska visas:

Om frågan Har du utfodrat (nöd- eller stödutfodrat) dina renar i din vintergruppen vid något tillfälle under de senaste fem åren? Om samebyn inte är uppdelad i olika vintergrupper svarar du för hela samebyn. Obs: här avses utfodring av vintergrupp (slakt- och livren) i hägn och/eller på fribete i över två veckor, men ej under flytt och samling som pågår under en kortare period än två veckor. innehåller något av dessa svarsalternativ

- Ja

123) Om du använt mineral, ange märke och typ av mineral som du senast använt.

I hägn:

På fribete:

Denna informationsbox visas endast i läge förhandsgranskningen.

Följande kriterium måste vara uppfyllda för att följande fråga ska visas:

Om frågan Har du utfodrat (nöd- eller stödutfodrat) dina renar i din vintergruppen vid något tillfälle under de senaste fem åren? Om samebyn inte är uppdelad i olika vintergrupper svarar du för hela samebyn. Obs: här avses utfodring av vintergrupp (slakt- och livren) i hägn och/eller på fribete i över två veckor, men ej under flytt och

samling som pågår under en kortare period än två veckor. innehåller något av dessa svarsalternativ

- Ja

4. Frågor om övriga fodermedel och rutiner vid utfodring under de senaste fem åren.

124) Hur vanligt förekommande är det att renarna ges tillgång till saltsten vid utfodring (tänk på de senaste fem åren)? Välj ett/flera alternativ.

	I hägn	På fribete
I princip alltid vid utfodring (anges som enda svarsalternativ)	☐	☐
Periodvis vid utfodring	☐	☐
Enstaka säsonger	☐	☐
Aldrig (anges som enda svarsalternativ)	☐	☐
Under särskilda omständigheter (exempelvis till renar i sämre kondition och i sjukhägn)	☐	☐
Annat, ange i kommentarer nedan	☐	☐

Denna informationsbox visas endast i läge förhandsgranskningen.

Följande kriterium måste vara uppfyllda för att följande fråga ska visas:

Om frågan Har du utfodrat (nöd- eller stödutfodrat) dina renar i din vintergruppen vid något tillfälle under de senaste fem åren? Om samebyn inte är uppdelad i olika vintergrupper svarar du för hela samebyn. Obs: här avses utfodring av vintergrupp (slakt- och livren) i hägn och/eller på fribete i över två veckor, men ej under flytt och samling som pågår under en kortare period än två veckor. innehåller något av dessa svarsalternativ

- Ja

125) Ange övriga fodermedel som använts under de senaste fem åren (till exempel foderjäst).

I hägn

På fribete

Denna informationsbox visas endast i läge förhandsgranskningen.

Följande kriterium måste vara uppfyllda för att följande fråga ska visas:

Om frågan Har du utfodrat (nöd- eller stödutfodrat) dina renar i din vintergruppen vid något tillfälle under de senaste fem åren? Om samebyn inte är uppdelad i olika vintergrupper svarar du för hela samebyn. Obs: här avses utfodring av vintergrupp (slakt- och livren) i hägn och/eller på fribete i över två veckor, men ej under flytt och samling som pågår under en kortare period än två veckor. innehåller något av dessa svarsalternativ

- Ja

126) Plats för kommentarer:

Denna informationsbox visas endast i läge förhandsgranskningen.

Följande kriterium måste vara uppfyllda för att följande fråga ska visas:

Om frågan Har du utfodrat (nöd- eller stödutfodrat) dina renar i din vintergruppen vid något tillfälle under de senaste fem åren? Om samebyn inte är uppdelad i olika vintergrupper svarar du för hela samebyn. Obs: här avses utfodring av vintergrupp (slakt- och livren) i hägn och/eller på fribete i över två veckor, men ej under flytt och samling som pågår under en kortare period än två veckor. innehåller något av dessa svarsalternativ

- Ja

eller

Om frågan 2015/2016 innehåller något av dessa svarsalternativ

- Utfodrade i hägn

eller

Om frågan 2016/2017 innehåller något av dessa svarsalternativ

- Utfodrade i hägn

eller

Om frågan 2017/2018 innehåller något av dessa svarsalternativ

- Utfodrade i hägn

eller

Om frågan 2018/2019 innehåller något av dessa svarsalternativ

- Utfodrade i hägn

eller

Om frågan 2019/2020 innehåller något av dessa svarsalternativ

- Utfodrade i hägn

eller

Om frågan 2020/2021 innehåller något av dessa svarsalternativ

- Utfodrade i hägn

4. Frågor om övriga fodermedel och rutiner vid utfodring under de senaste fem åren.

127) Ange vattentillgång vid utfodring i hägn under de senaste fem åren? Välj ett/flera alternativ.

☐Fri tillgång på snö
☐Snö i krubba/balja
☐Vatten i krubba/balja
☐Bäck
☐Kallkälla
☐Annat, ange i kommentarfältet nedan.

Denna informationsbox visas endast i läge förhandsgranskningen.

Följande kriterium måste vara uppfyllda för att följande fråga ska visas:

Om frågan Har du utfodrat (nöd- eller stödutfodrat) dina renar i din vintergruppen vid något tillfälle under de senaste fem åren? Om samebyn inte är uppdelad i olika vintergrupper svarar du för hela samebyn. Obs: här avses utfodring av vintergrupp (slakt- och livren) i hägn och/eller på fribete i över två veckor, men ej under flytt och samling som pågår under en kortare period än två veckor. innehåller något av dessa svarsalternativ

- Ja

eller

Om frågan 2015/2016 innehåller något av dessa svarsalternativ

- Utfodrade i hägn

eller

Om frågan 2016/2017 innehåller något av dessa svarsalternativ

- Utfodrade i hägn

eller

Om frågan 2017/2018 innehåller något av dessa svarsalternativ

- Utfodrade i hägn

eller

Om frågan 2018/2019 innehåller något av dessa svarsalternativ

- Utfodrade i hägn

eller

Om frågan 2019/2020 innehåller något av dessa svarsalternativ

- Utfodrade i hägn

128) Plats för kommentarer:

Denna informationsbox visas endast i läge förhandsgranskningen.

Följande kriterium måste vara uppfyllda för att följande fråga ska visas:

Om frågan Har du utfodrat (nöd- eller stödutfodrat) dina renar i din vintergruppen vid något tillfälle under de senaste fem åren? Om samebyn inte är uppdelad i olika vintergrupper svarar du för hela samebyn. Obs: här avses utfodring av vintergrupp (slakt- och livren) i hägn och/eller på fribete i över två veckor, men ej under flytt och samling som pågår under en kortare period än två veckor. innehåller något av dessa svarsalternativ

- Ja

4. Frågor om övriga fodermedel och rutiner vid utfodring under de senaste fem åren.

129) Finns det rutiner för tillvänjning av fodret?

○ Ja

Nej

Denna informationsbox visas endast i läge förhandsgranskningen.

Följande kriterium måste vara uppfyllda för att följande fråga ska visas:

Om frågan Har du utfodrat (nöd- eller stödutfodrat) dina renar i din vintergruppen vid något tillfälle under de senaste fem åren? Om samebyn inte är uppdelad i olika vintergrupper svarar du för hela samebyn. Obs: här avses utfodring av vintergrupp (slakt- och livren) i hägn och/eller på fribete i över två veckor, men ej under flytt och samling som pågår under en kortare period än två veckor. innehåller något av dessa svarsalternativ

- Ja

130) Plats för kommentarer:

Denna informationsbox visas endast i läge förhandsgranskningen.

Följande kriterium måste vara uppfyllda för att följande fråga ska visas:

Om frågan Har du utfodrat (nöd- eller stödutfodrat) dina renar i din vintergruppen vid något tillfälle under de senaste fem åren? Om samebyn inte är uppdelad i olika vintergrupper svarar du för hela samebyn. Obs: här avses utfodring av vintergrupp (slakt- och livren) i hägn och/eller på fribete i över två veckor, men ej under flytt och samling som pågår under en kortare period än två veckor. innehåller något av dessa svarsalternativ

- Ja

4. Frågor om övriga fodermedel och rutiner vid utfodring under de senaste fem åren.

131) * Tar du vanligtvis bort överblivet foder innan du utfodrar med nytt?

Ja

Nej

Denna informationsbox visas endast i läge förhandsgranskningen.

Följande kriterium måste vara uppfyllda för att följande fråga ska visas:

Om frågan Har du utfodrat (nöd- eller stödutfodrat) dina renar i din vintergruppen vid något tillfälle under de senaste fem åren? Om samebyn inte är uppdelad i olika vintergrupper svarar du för hela samebyn. Obs: här avses utfodring av vintergrupp (slakt- och livren) i hägn och/eller på fribete i över två veckor, men ej under flytt och samling som pågår under en kortare period än två veckor. innehåller något av dessa svarsalternativ

- Ja

132) Plats för kommentarer:

Denna informationsbox visas endast i läge förhandsgranskningen.

Följande kriterium måste vara uppfyllda för att följande fråga ska visas:

Om frågan Har du utfodrat (nöd- eller stödutfodrat) dina renar i din vintergruppen vid något tillfälle under de senaste fem åren? Om samebyn inte är uppdelad i olika vintergrupper svarar du för hela samebyn. Obs: här avses utfodring av vintergrupp (slakt- och livren) i hägn och/eller på fribete i över två veckor, men ej under flytt och samling som pågår under en kortare period än två veckor. innehåller något av dessa svarsalternativ

- Ja

och

Om frågan Tar du vanligtvis bort överblivet foder innan du utfodrar med nytt? innehåller något av dessa svarsalternativ

- Ja

4. Frågor om övriga fodermedel och rutiner vid utfodring under de senaste fem åren.

133) Var tömmer du vanligtvis överblivet foder?

- I hagen
- Utanför hagen
- Annat, ange i kommentarsfältet nedan.

Denna informationsbox visas endast i läge förhandsgranskningen.

Följande kriterium måste vara uppfyllda för att följande fråga ska visas:

Om frågan Har du utfodrat (nöd- eller stödutfodrat) dina renar i din vintergruppen vid något tillfälle under de senaste fem åren? Om samebyn inte är uppdelad i olika vintergrupper svarar du för hela samebyn. Obs: här avses utfodring av vintergrupp (slakt- och livren) i hägn och/eller på fribete i över två veckor, men ej under flytt och samling som pågår under en kortare period än två veckor. innehåller något av dessa svarsalternativ

- Ja

och

Om frågan Tar du vanligtvis bort överblivet foder innan du utfodrar med nytt? innehåller något av dessa svarsalternativ

- Ja

134) Plats för kommentarer:

Denna informationsbox visas endast i läge förhandsgranskningen.

Följande kriterium måste vara uppfyllda för att följande fråga ska visas:

Om frågan Har du utfodrat (nöd- eller stödutfodrat) dina renar i din vintergruppen vid något tillfälle under de senaste fem åren? Om samebyn inte är uppdelad i olika vintergrupper svarar du för hela samebyn. Obs: här avses utfodring av vintergrupp (slakt- och livren) i hägn och/eller på fribete i över två veckor, men ej under flytt och samling som pågår under en kortare period än två veckor. innehåller något av dessa svarsalternativ

- Ja

Strax klart, här följer de sista frågorna om utfodring:

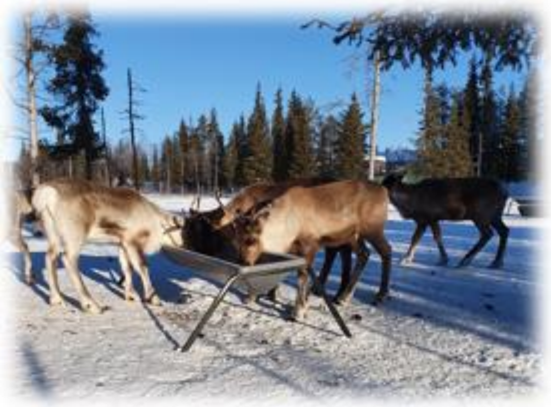

135) Påverkar tillgången av olika fodermedel (ensilage/pellets/lav) ditt val av foder? Förklara gärna i kommentarsfältet nedan.

- Ja, ange hur i kommentarsfältet nedan
- Nej

Denna informationsbox visas endast i läge förhandsgranskningen.

Följande kriterium måste vara uppfyllda för att följande fråga ska visas:

Om frågan Har du utfodrat (nöd- eller stödutfodrat) dina renar i din vintergruppen vid något tillfälle under de senaste fem åren? Om samebyn inte är uppdelad i olika vintergrupper svarar du för hela samebyn. Obs: här avses utfodring av vintergrupp (slakt- och livren) i hägn och/eller på fribete i över två veckor, men ej under flytt och samling som pågår under en kortare period än två veckor. innehåller något av dessa svarsalternativ

- Ja

136) Efterfrågar du analys av grovfodret med avseende på näringsinnehåll och/eller hygien?

- Ja, näringsinnehåll
- Ja, hygien
- Ja, både näringsinnehåll och hygien
- Nej

Denna informationsbox visas endast i läge förhandsgranskningen.

Följande kriterium måste vara uppfyllda för att följande fråga ska visas:

Om frågan Har du utfodrat (nöd- eller stödutfodrat) dina renar i din vintergruppen vid något tillfälle under de senaste fem åren? Om samebyn inte är uppdelad i olika vintergrupper svarar du för hela samebyn. Obs: här avses utfodring av vintergrupp (slakt- och livren) i hägn och/eller på fribete i över två veckor, men ej under flytt och samling som pågår under en kortare period än två veckor. innehåller något av dessa svarsalternativ

- Ja

137) Plats för kommentarer:

Denna informationsbox visas endast i läge förhandsgranskningen.

Följande kriterium måste vara uppfyllda för att följande fråga ska visas:

Om frågan Har du utfodrat (nöd- eller stödutfodrat) dina renar i din vintergruppen vid något tillfälle under de senaste fem åren? Om samebyn inte är uppdelad i olika vintergrupper svarar du för hela samebyn. Obs: här avses utfodring av vintergrupp (slakt- och livren) i hägn och/eller på fribete i över två veckor, men ej under flytt och samling som pågår under en kortare period än två veckor. innehåller något av dessa svarsalternativ

- Ja

138) Lämna gärna övriga kommentarer kopplade till utfodring och/eller övriga reflektioner på enkäten här:

Denna informationsbox visas endast i läge förhandsgranskningen.

Dessa actions kommer att genomföras vid följande alternativ:
Ja, jag vill veta mera! : Vidarebefordra till en extern URL (https://response.questback.com/statensveterinrmedicinskaansta/erfarenheteravsjukdomigaochmunhosren)

Var med och bidra till mera kunskap om renhälsa och utfodring!

Vi söker deltagare som vill vara med i en planerad fördjupad intervju rörande renhälsa och effekter av utfodring. Intervjun kommer i huvudsak att genomföras av Karin Wallin Philippot i Sverige och av Alfa Josteinsdottir i Norge, där vi tillsammans kommer överens om en tid och plats för genomförandet.

139) * Vill du vara med? Klicka JA för få mera information i ett separat enkätblad.

- Nej tack
- Ja, jag vill veta mera!

© Copyright www.questback.com. All Rights Reserved.
Trial Essentials for free - Click here to create your survey today.
