# Supplementary material for: Infectious keratoconjunctivitis in semi-domesticated reindeer (Rangifer tarandus tarandus): a questionnaire-based study among reindeer herders in Norway and Sweden
Source: Acta Vet Scand. 2023 Jul 12;65:34. doi: 10.1186/s13028-023-00694-x (PMC10337086; doi:10.1186/s13028-023-00694-x)

Additional file 4 Specified herd size per reindeer herding region from 76 respondents, 33 from Norway and 43 from Sweden, given in the questionnaire survey given to herders regarding health and supplementary feeding of semi-domesticated reindeer. In Sweden: Norrbotten, Västerbotten and Dalarna/Jämtland, and in Norway: Øst-Finnmark, Vest Finnmark, Troms, Nordland, Nord-Trøndelag, and Sør-Trøndelag and Hedmark

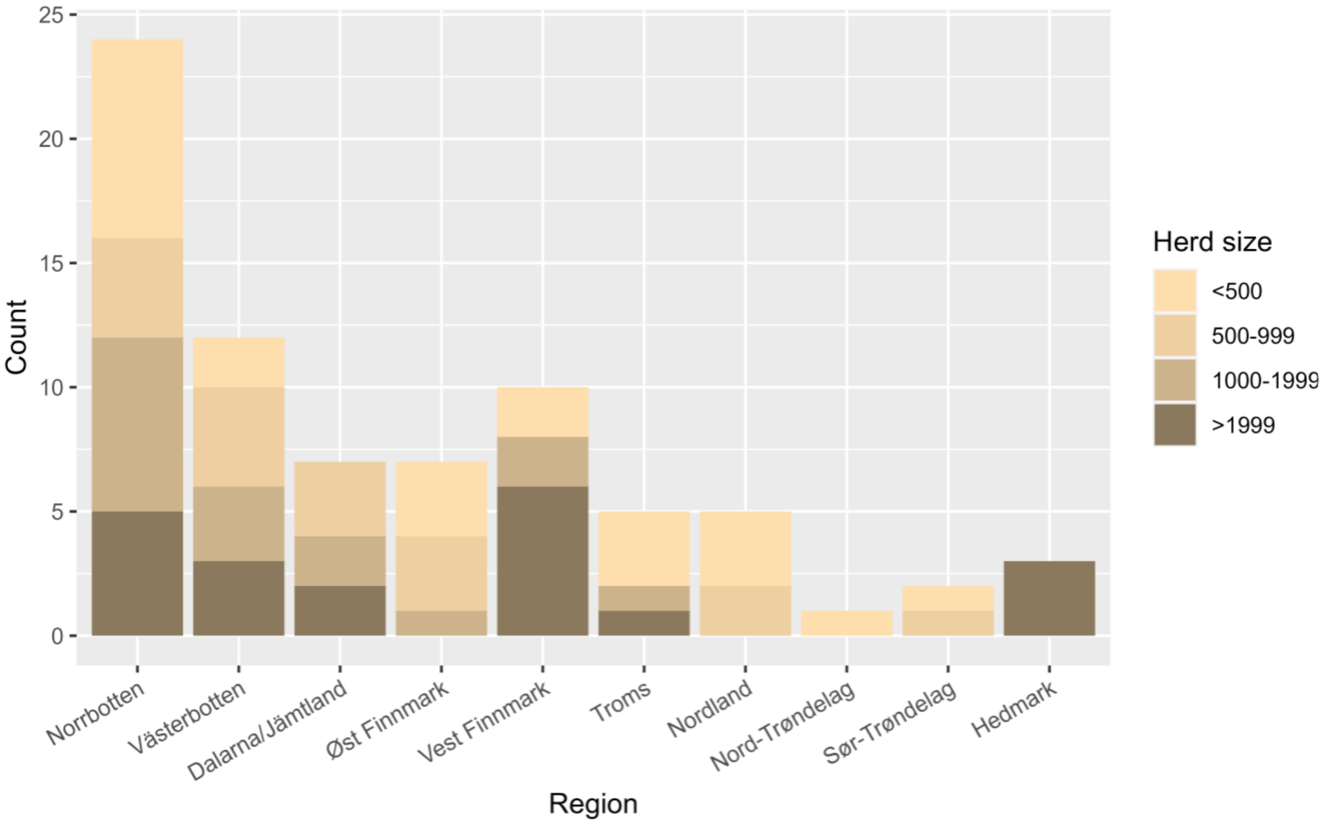

Supplement: Supplementary file 4 — Additional file 4: Specified herd size per reindeer herding region in each country from 76 respondents, 33 from Norway and 43 from Sweden. [file 13028_2023_694_MOESM4_ESM.pdf]
